# Recent Trends and Challenges on the Non-Targeted Analysis and Risk Assessment of Migrant Non-Intentionally Added Substances from Plastic Food Contact Materials

**DOI:** 10.3390/toxics13070543

**Published:** 2025-06-28

**Authors:** Pablo Miralles, Esther Fuentes-Ferragud, Cristina Socas-Hernández, Clara Coscollà

**Affiliations:** 1 Foundation for the Promotion of Health and Biomedical Research of the Valencian Community (FISABIO), FISABIO-Public Health, Av. Catalunya 21, 46020 Valencia, Spain; esther.fuentes@fisabio.es (E.F.-F.); asocashe@ull.edu.es (C.S.-H.); clara.coscolla@fisabio.es (C.C.); 2 Environmental and Public Health Analytical Chemistry, Research Institute for Pesticides and Water, University Jaume I, Av. Sos Baynat s/n, 12071 Castelló de la Plana, Spain; 3 Departamento de Química, Unidad Departamental de Química Analítica, Facultad de Ciencias, Universidad de La Laguna (ULL), Avda. Astrofísico Fco. Sánchez s/n, 38206 San Cristóbal de La Laguna, Spain; 4 Instituto Universitario de Enfermedades Tropicales y Salud Pública de Canarias, Universidad de La Laguna (ULL), Avda. Astrofísico Fco. Sánchez s/n, 38206 San Cristóbal de La Laguna, Spain

**Keywords:** food contact materials, migration, non-intentionally added substances, non-target analysis, organic contaminants, plastics, untargeted screening

## Abstract

Non-intentionally added substances (NIAS) in plastic food contact materials represent a critical undercharacterized chemical safety concern, caused by their inherent diversity, potential toxicity, and regulatory challenges. This review synthesizes recent advances and persistent gaps in NIAS analysis, with a primary focus on analytical workflows for non-targeted analysis, alongside a consideration of risk assessment and toxicological prioritization frameworks. Conventional plastics (e.g., polyethylene, polypropylene, or polyethylene terephthalate) as well as emerging materials (e.g., bioplastics and recycled polymers) exhibit different NIAS profiles, including oligomers, degradation products, additives, and contaminants, requiring specific approaches for migration testing, extraction, and detection. Advanced techniques, such as ultra-high-performance liquid chromatography or two-dimensional gas chromatography coupled with high-resolution mass spectrometry, have enabled non-targeted analysis approaches. However, the field remains constrained by spectral library gaps, limited reference standards, and inconsistent data processing protocols, resulting in heavy reliance on tentative identifications. Risk assessment procedures mainly employ the Threshold of Toxicological Concern and classification by Cramer’s rules. Nevertheless, addressing genotoxicity, mixture effects, and novel hazards from recycled or bio-based polymers remains challenging with these approaches. Future priorities and efforts may include expanding spectral databases, harmonizing analytical protocols, and integrating in vitro bioassays with computational toxicology to refine hazard characterization.

## 1. Introduction

Food contact materials (FCMs) are widely used in the food industry to package and store products, playing a critical role in ensuring product stability, extending shelf life, and preserving food quality. To achieve these objectives, FCMs are formulated with a variety of intentionally added substances (IAS), such as monomers, plasticizers, antioxidants, lubricants, UV absorbents, and stabilizers [1,2]. However, during production, use, or recycling, FCMs may also contain non-intentionally added substances (NIAS) that are not purposefully included in the formulation. NIAS can arise from several sources, including the degradation of polymers or additives, reaction byproducts resulting from incomplete polymerization or improperly cured adhesives, impurities present in the raw materials, and contaminants introduced during recycling processes or through inappropriate consumer use [2,3]. The complexity of these origins underscores the analytical challenges in identifying and quantifying NIAS, which can migrate into food and potentially pose health risks. The presence of NIAS in FCMs represents a dual challenge. On the one hand, the potential migration of these substances into food poses a human health risk, particularly when the toxicological properties of the NIAS are unknown or poorly characterized [1]. This uncertainty can hinder risk assessments and regulatory decision-making, especially when dealing with genotoxic, endocrine-disrupting, or bioaccumulative substances for which no safety thresholds are established. On the other hand, the chemical diversity and typically low concentration of NIAS make their identification and quantification an analytical challenge. Traditional targeted analytical methods, which rely on prior knowledge of the analytes and the availability of reference standards, are often insufficient to capture the wide array of NIAS present in complex matrices [4]. Consequently, the scientific community has increasingly turned to non-targeted analysis (NTA) approaches, such as non-targeted identification and suspect screening, employing advanced high-resolution mass spectrometry (HRMS) to detect and characterize compounds without prior knowledge about their presence [5,6].

Regulatory frameworks have evolved in tandem with these analytical advancements to ensure food safety. The European Commission Regulation (EU) No. 10/2011 [7], which was recently amended by Commission Regulation (EU) 2025/351 [8], for instance, establishes specific migration limits for substances in plastic FCMs and mandates that manufacturers assess the potential migration of both IAS and NIAS under worst-case conditions using appropriate food simulants. These regulations require comprehensive migration testing, which involves exposing FCMs to food simulants under controlled conditions that mimic real-world scenarios. However, unlike IAS, NIAS are often not individually regulated, and their assessment relies on general safety principles rather than substance-specific limits. Such regulatory incompleteness underscores the need for robust analytical and toxicological approaches to evaluate potential risks. This makes rigorous testing even more essential to confirm that any migrating substances remain within established safety limits, thereby protecting consumer health and maintaining public trust in food safety standards [7,8].

The integration of ultra-high-performance liquid chromatography (UHPLC) with HRMS has enabled the simultaneous detection and structural elucidation of a multitude of compounds at trace levels. Techniques such as quadrupole time-of-flight (QTOF) and Orbitrap mass spectrometry have provided the sensitivity, selectivity, and mass accuracy necessary for non-targeted approaches, facilitating the detection of compounds that might otherwise remain undetected [9,10]. Moreover, innovations in data acquisition modes, such as data-independent acquisition (DIA) and data-dependent acquisition (DDA), have allowed for the collection of comprehensive spectral data, which can then be processed using advanced computational tools and spectral databases. These developments have not only improved the detection capabilities but also enhanced the reliability of compound identification, even in the absence of reference standards [11,12].

The analytical challenges associated with NIAS detection are multifaceted. Sample preparation, for example, is a critical step that can influence the overall sensitivity and accuracy of the analysis. Migration tests must be carefully designed to mimic the worst-case conditions of FCM use, employing a range of food simulants that reflect different food types [13]. The extraction and concentration of migrants from these simulants require optimized procedures to ensure that NIAS are not lost or degraded during analysis [14]. In addition, the diverse chemical properties of NIAS, from volatile to non-volatile compounds, demand the use of complementary chromatographic techniques. While liquid chromatography (LC) is often preferred for non-volatile compounds due to its compatibility with soft ionization methods such as electrospray ionization (ESI), gas chromatography (GC) is typically employed for the analysis of volatile and semi-volatile substances using electron ionization (EI) [15,16].

The shift toward NTA approaches represents a paradigm change in the analysis of FCMs. Unlike traditional targeted approaches, NTA does not require a predefined list of target compounds, thereby enabling the detection of a broader spectrum of substances. This is particularly important given the sheer number of potential NIAS, many of which may arise from unexpected sources or novel degradation pathways. The comprehensive nature of non-targeted screening has revealed a far greater chemical complexity in FCMs than previously recognized, highlighting the need for ongoing research and method development [12,17]. In addition to technical advancements, recent studies have also focused on the integration of chemometric techniques to interpret complex datasets generated by non-targeted screening. Multivariate statistical methods, such as principal component analysis and partial least-squares discriminant analysis, have been applied to differentiate between various FCM formulations and to identify key markers that may be indicative of NIAS presence [11,18]. These approaches enable a deeper understanding of the factors influencing NIAS migration, including the type of polymer, the nature of additives, and the conditions of plastic use and recycling. Furthermore, by highlighting patterns and clustering substances with similar occurrence profiles or migration behaviors, chemometric tools can support prioritization by flagging recurring or highly migratory unknowns for further toxicological evaluations. When combined with metadata such as polymer origin or known use categories, these statistical models can help focus risk assessment efforts on substances of potential concern.

Understanding the challenges associated with the migration of NIAS from plastic FCMs and their implications for food safety is a current matter of concern. As research progresses, there is a growing recognition of the necessity for advanced analytical methodologies to address the complexities posed by NIAS [19]. The study of NIAS in plastic FCMs is relevant for several reasons: (a) Integrated analytical and toxicological approaches are required to comprehensively assess potential risks. (b) The growth of NTA techniques has enabled the detection and identification of a much wider range of compounds. (c) By detecting new unknown compounds, the understanding of chemical migration and potential human exposure risks is increased. (d) The integration of advanced analytical techniques with evolving regulatory frameworks highlights the essential role of scientific innovation in public health protection. This review primarily focuses on analytical strategies for the NTA of NIAS in plastic FCMs, complemented by a brief outlook on toxicological prioritization frameworks. Broader aspects such as risk communication and policy development, though highly relevant, are beyond the scope of this article.

## 2. Literature Search and Reviewed Studies

This review article focuses on recent research (published since 2018) that employs NTA approaches for the analysis of NIAS migrating from plastic or polymer FCMs. The selection process was based on literature searches of the Web of Science Core Collection using keywords such as “non-intentionally added substances”, “food contact materials”, “non-target analysis”, “untargeted analysis”, and “untargeted screening”, among other related keywords. Only original research articles were considered; review articles, book chapters, studies focused solely on targeted analysis, or those unrelated to food contact polymers were excluded. The search results were meticulously examined, and 54 relevant studies on this topic were selected and summarized (Table 1). While the review aims to provide an overview of the current state of the art in this rapidly evolving field, it is acknowledged that some pertinent studies may not have been included. In summary, this review article seeks to provide a detailed and critical examination of recent trends and challenges in the NTA of NIAS migrating from plastic and polymer FCMs by integrating information from the scientific literature and selected regulatory documents, including EU Regulation 10/2011 and its amendments [7,8], EFSA scientific opinions, and FDA guidelines. These documents are referenced primarily to contextualize analytical requirements and regulatory expectations relevant to NIAS identification.

## 3. Studied Polymers and Food Contact Materials

The research articles summarized in Table 1 examine a diverse range of polymeric matrices employed in FCMs, including conventional and recycled plastics, emerging biodegradable materials, as well as composite systems that incorporate multilayer structures, adhesives, or coatings. This diversity reflects both current trends in FCM production and key areas of regulatory and toxicological concern. While not exhaustive, the polymer types covered are representative of materials with high market prevalence and/or growing relevance due to sustainability goals (e.g., recycled and bio-based plastics), thereby including substances most likely to contribute to consumer exposure.

Polyethylene terephthalate (PET) is among the most frequently studied polymers, with investigations including virgin and recycled PET (rPET) in bottled water [24], microwaveable containers [25], and beverage bottles [39]. The prevalence of PET in single-use packaging drives research into its oligomer migration, particularly cyclic and linear terephthalate derivatives [59], with studies emphasizing rPET due to concerns over contaminants introduced during reprocessing [62]. Similarly, polyethylene (PE), including high-density PE (HDPE), low-density PE (LDPE), and recycled PE (rPE) and LDPE (rLDPE), has been extensively examined in films, reusable bottles, and multilayer packaging [50,64,67]. These studies often focus on antioxidant degradation products, plasticizers like phthalates, and hydrocarbon oligomers, highlighting the susceptibility of the material to releasing NIAS under thermal stress as a consequence of recycling processes [18,42]. However, the ability of current NTA methodologies to fully capture NIAS arising from recycling remains limited due to the higher complexity and variability of recycled matrices, the presence of trace-level contaminants from previous use cycles, and the lack of comprehensive spectral libraries or reference standards for unknown degradation or contamination products.

Polypropylene (PP) is another widely analyzed polymer, featured in food storage containers, baby bottles, and microwavable trays [35,43,54]. Its applications at high temperatures, such as microwave heating or dishwashing, have prompted investigations into additive degradation and the formation of volatile aldehydes or phenolic compounds [36,54]. Recycled PP (rPP) has also been scrutinized for contaminants arising from post-consumer waste, including odoriferous hydrocarbons and surfactant residues [48]. These findings underscore the need for advanced analytical methods to address the complexity of NIAS in recycled FCMs.

Biopolymers, such as polylactic acid (PLA) and polyester (PES) mixtures, represent an emerging focus as a result of their growing use as eco-friendly alternatives. Studies on PLA-based films and pellets reveal cyclic lactide oligomers and adipate derivatives as predominant migrants, with migration tests simulating both aqueous and fatty food conditions [6,60,61].

Epoxy-based coatings, commonly applied to metal cans, have been extensively evaluated for NIAS derived from resin precursors like bisphenol derivatives, such as Bisphenol A diglycidyl ether (BADGE), and their reaction byproducts [40,41]. These coatings, which are designed for durability, can potentially release complex oligomers and phenolic compounds under migration conditions [38]. Similarly, polyurethane (PUR) and polyester–phenolic coatings in metallic FCMs have been linked to isocyanate and cyclic oligomer migration, underscoring the challenges of identifying reactive intermediates in polymer matrices [44,45,55].

Kitchenware and reusable food utensils, which are often fabricated from polyamide (PA), polybutylene terephthalate (PBT), or melamine–formaldehyde resins, have been studied for their migration of monomers, plasticizers, and oligomers under simulated cooking conditions [21,33,34]. For example, PA-based kitchen tools release adipate plasticizers and cyclic oligomers, while melamine resins leach formaldehyde and melamine monomers, particularly after UV exposure [34].

Specific applications, such as UV-cured varnishes on printed PP films, introduce unique NIAS like photoinitiators (e.g., benzophenone) and ester monomers, which migrate into ethanol-based food simulants [5]. Similarly, multilayer packaging materials, combining PE and PA, have been studied, showing the migration of caprolactam and cyclic oligomers and reflecting the interplay between polymer layers and migration pathways [30]. The analysis of plant fiber/plastic composites (PPCs) further expands the scope, revealing migrants such as pesticides and melamine derivatives that likely originate from natural fiber additives or adhesives [69]. However, these hybrid materials pose additional challenges for NIAS analysis due to limited regulatory guidance specific to composite systems, complicating both the identification and risk assessment of NIAS in PPCs.

While PET, PE, and PP are well-studied in the literature due to their ubiquity in packaging, emerging materials like biopolymers and composites introduce new analytical complexities. In fact, recycled polymers demand particular attention due to their heterogeneous NIAS profiles. Each polymer type presents distinct analytical challenges: for instance, PET and PES may generate thermally labile or semi-volatile degradation products, while PE and PP matrices often produce low-polarity oligomers that are difficult to ionize or extract. Composite and bio-based materials may also introduce matrix effects or interferents from natural additives and coatings. Thus, tailored migration studies considering both specific scenarios of use (e.g., baby bottles and microwavable containers) and everyday items (e.g., water bottles and candy wrappers) are required to ensure consumer safety.

## 4. Sample Preparation

Sample preparation is a critical step in the non-targeted screening of NIAS from plastic FCMs, as it directly influences the detection sensitivity, accuracy, and relevance of detected compounds. Inadequate or poorly optimized preparation can lead to false negatives, analyte loss, or biased compound profiles, ultimately compromising the reliability of NIAS identification. This process typically involves two complementary approaches: (i) migration tests using food simulants to replicate real food contact scenarios, according to Regulation (EU) 10/2011 [7], and (ii) extraction techniques, such as solvent extraction and total dissolution–precipitation methods, to isolate migrants from the polymer matrix or from migration solutions. The choice of the extraction method depends on the research objectives: to assess compliance with a regulatory specific migration limit (SML), simulate consumer exposure to NIAS, or characterize the chemical landscape of FCMs. Among the reviewed studies, some prioritized exposure simulations through standardized migration protocols, while others focused on exploratory chemical profiling via exhaustive extraction methods. Despite the diversity of protocols, there is a growing need for methodological harmonization to improve comparability and risk-based interpretation.

### 4.1. Migration Tests Using Food Simulants

**Figure 1:**
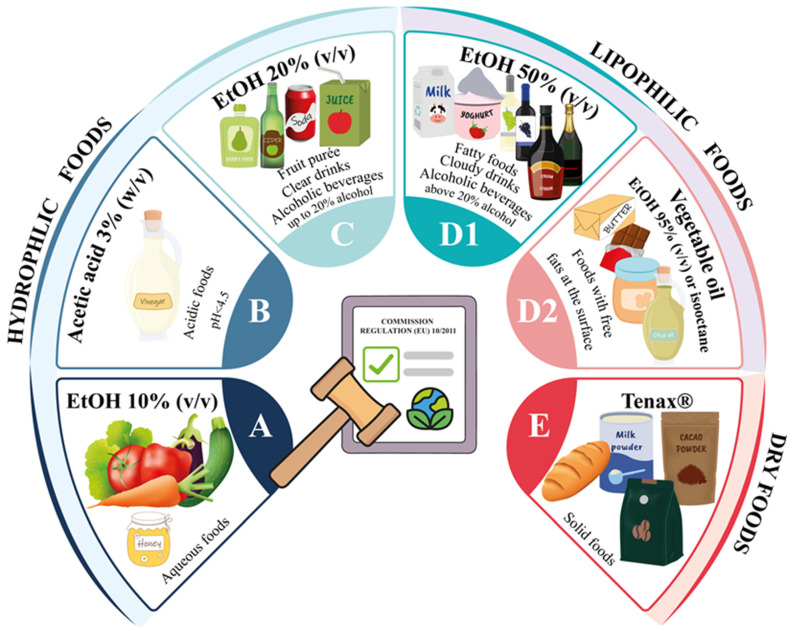
Common food simulants and their applications for migration testing of plastic FCMs.

Migration testing under controlled laboratory conditions remains the most common practice for evaluating the release of substances from plastic FCMs into food, as established by Regulation (EU) 10/2011 [7] and its recent amendment [8]. This regulation defines standardized food simulants (e.g., aqueous, acidic, alcoholic, or fatty substitutes) and testing conditions (time and temperature) tailored to the intended use of the material. Among the 54 reviewed studies in Table 1, migration tests were widely employed to simulate both routine and extreme scenarios, such as long-term storage, microwave heating, or dishwashing. A summary of the most common food simulants and their applications in the reviewed studies is depicted in Figure 1. However, despite their regulatory value, food simulants may not fully replicate real food matrices and can sometimes lead to the over- or underestimation of actual migration, particularly for hydrophobic or reactive compounds.

For aqueous and acidic foods (simulants A: 10% ethanol (EtOH), B: 3% acetic acid, and C: 20% EtOH, all in water solution), studies often applied conditions of 40–60 °C for 10 days to mimic long-term storage [20,63]. For instance, Díaz-Galiano et al. [25] tested PP microwavable containers with simulant A at 40 °C for 10 days to reflect typical reheating practices. In the study conducted by Vera et al. [66], ethylene–vinyl acetate (EVA) corks, intended to be used as sealants for wine bottles, were exposed to simulants B and C at 60 °C for 10 days.

Fatty food simulants (D1: 50% EtOH in water; D2: vegetable oil and its substitutes and 95% EtOH or isooctane) are also frequently used. For example, Radusin et al. [50] performed migration testing of multilayer PE films, including rLDPE, with simulant D2 (95% EtOH) at 20, 40, and 60 °C for 10 days to evaluate the migration of different chemical compounds, such as antioxidants and oligomers, from food packaging into fatty foods. High-temperature migration testing was applied to PA kitchenware, such as soup spoons, using food simulant D2 (95% EtOH) at 70 °C for 2 h to simulate cooking and use conditions [33]. Similarly, styrene–acrylonitrile copolymer (SAN) and acrylonitrile–butadiene–styrene (ABS) kitchenware were tested at 70–100 °C for 2 h using different simulants in the work conducted by Kubicova et al. [37].

Regulation (EU) 10/2011 also outlines specific protocols for repeated-use articles [7]. For example, baby feeding bottles have been tested with food simulant D1 at 70 °C for 2 h to simulate repeated sterilization cycles and NIAS migration into hot milk [26,43]. Similarly, reusable plastic bottles made from PE, polycarbonate (PC), and other polymers were tested with water at room temperature for 24–48 h to model daily use, revealing the migration of oligomers and slip agents even after dishwashing [57,58]. Microwave and oven exposure, though not explicitly detailed in Regulation (EU) 10/2011, were incorporated into migration protocols to address real heating scenarios. For instance, microwave trays and oven bags were tested at 700 W for 5 min (max 102 °C) using simulant D2 (95% EtOH), showing higher migration levels of photoinitiators and plasticizers compared to conventional oven heating [52].

Migration into dry foods, using food simulant E (Tenax^®^, Verona, Italy), was tested on polyolefin films and multilayer packaging. For example, Tenax migration at 60 °C for 10 days effectively captured volatile NIAS like aldehydes and hydrocarbons from PP and PE films [65]. Similarly, in the work conducted by García-Ibarra et al. [29], migration from several plastic films composed of PE, PET, PA, PP, and EVA was tested using simulant E at 60 °C for 10 days to identify potential migrants in packaging.

For PPCs, migration into 4% acetic acid (similar to food simulant B) at 60 °C for 2 h under an ultrasonic bath was employed to mimic contact with acidic foods, revealing migrants like pesticides and melamine derivatives [69]. rPET pellets were tested with water at 40 °C for 10 days, revealing acetaldehyde migration, a key indicator of potential polymer degradation during recycling [62]. It should be noted that migration testing may affect NIAS identification confidence due to dilution effects or matrix interferences from simulants, particularly in cases of low-abundance or reactive compounds.

### 4.2. Extraction Techniques

Extraction techniques are essential for isolating NIAS from plastic FCMs or migration solutions, particularly when migration tests alone are not sufficient to mimic the full chemical complexity of polymer components. The reviewed studies employed diverse methods, including solvent extraction, total dissolution–precipitation methods, and solid-phase microextraction (SPME), each with distinct advantages and limitations in terms of efficiency, selectivity, and compatibility with analytical platforms.

Solvent extraction remains the most widely used method for extracting NIAS from plastic matrices, using organic solvents to release embedded additives, oligomers, or degradation products. However, solvent selection involves trade-offs between extraction comprehensiveness and selectivity, as well as environmental and analytical considerations. Acetonitrile (ACN) and dichloromethane (DCM) were frequently employed due to their ability to solubilize a broad range of substances from the polymer matrix. While DCM is effective for extracting non-polar compounds, its high volatility and toxicity limit its routine use. ACN offers broader polarity coverage but may be less efficient for extracting hydrophobic NIAS. For instance, PES and PES-PUR coatings in metal food cans were extracted with ACN at 40 °C for 24 h, obtaining a wide range of additives, oligomers, and oligoesters [23,44,45]. Similarly, PP films and candy wrappers were treated with ACN at 70 °C for 6–24 h, extracting n-alkanes (C21–C29), butylated hydroxytoluene (BHT) and its degradation products, plasticizers (e.g., phthalates), and phenolic compounds, among others [27,28]. While effective, these approaches may introduce extraction biases that influence the range of detectable NIAS, especially for compounds with extreme polarity or thermal sensitivity.

Ultrasonic-assisted extraction, as applied to SAN and ABS kitchenware, enhanced the recovery of styrene and acrylonitrile oligomers, as well as aromatic amines, using methanol (MeOH) at 50 °C for 30 min [37]. While ultrasonic extraction improves efficiency by facilitating compound release, the use of elevated temperatures and sonication energy may also increase the risk of thermal or mechanical degradation of sensitive NIAS. However, solvent extraction faces some challenges in polymer compatibility. For example, PE and PP usually require more aggressive solvents like DCM or n-hexane:acetone (ACE) mixtures to overcome their crystallinity, whereas polar polymers such as PA and PUR can be readily extracted with EtOH or ACN [31,46].

On the other hand, total dissolution–precipitation methods are particularly effective for obtaining oligomers and high-molecular-weight NIAS, which are often trapped within polymer networks. These techniques dissolve the polymer, usually in a halogenated solvent (e.g., hexafluoroisopropanol (HFIP) for PET or DCM for PLA), and precipitate it using a non-solvent (e.g., MeOH or EtOH), leaving oligomers and additives in the supernatant. For example, PET pellets and bottles were dissolved in HFIP at 40 °C for 24 h, followed by precipitation with MeOH at 4 °C for 1 h [32,59]. Brenz et al. [22] isolated PES oligomers by treatment for approximately 30 min in an ultrasonic bath using a mixture of HFIP and DCM and reprecipitation with 2-propanol. Similarly, PLA-based biopolymers were treated with DCM and precipitated with EtOH [6,60]. However, these harsh chemical treatments may alter the polymer matrix and potentially affect the stability of certain oligomers or NIAS, which should be taken into consideration when interpreting analytical results.

Headspace (HS)-SPME and immersion SPME are solvent-free techniques ideal for volatile and semi-volatile NIAS, minimizing matrix interference. HS-SPME, which absorbs vapors from heated samples, was widely used for analyzing migrants in PET bottles and polyolefin films. The choice of fiber coating plays a key role influencing selectivity. For instance, divinylbenzene (DVB)/carboxen (CAR)/polydimethylsiloxane (PDMS) fibers are frequently used, which can extract a wide range of volatile compounds due to their double polarity. For example, DVB/CAR/PDMS fibers were used to extract several volatiles and odorous compounds from recycled PP [48]. In the work conducted by Cincotta et al. [24], aldehydes (e.g., octanal, nonanal, decanal, etc.) and hydrocarbons were detected in bottled water also using a DVB/CAR/PDMS fiber at 60 °C, while Li et al. [39] identified 1,247 volatile organic compounds (VOCs) in rPET with a PDMS/DVB/Carbon Wide Range fiber at 110 °C. Immersion SPME, where the fiber is directly exposed to migration solutions, proved effective for less volatile compounds. In this regard, Song et al. [53] quantified migrant substances from expanded polystyrene (EPS) and recycled EPS containers by immersing a DVB/CAR/PDMS SPME fiber in the migration solutions obtained using food simulants A and B. However, the recovery efficiency in SPME can vary significantly depending on the physicochemical properties of the NIAS, such as volatility and polarity. Additionally, issues such as fiber saturation and competitive adsorption can limit extraction capacity and selectivity, potentially affecting quantification accuracy.

Therefore, the reviewed studies demonstrate that the choice of sample preparation method depends on the polymer type, the physicochemical properties of the NIAS under observation, and the intended application of the FCM. While migration tests provide regulatory compliance data under simulated use conditions, extraction techniques offer deeper insights into the chemical complexity of polymers, including oligomers, additives, and degradation products.

## 5. Instrumental Analysis

**Figure 2:**
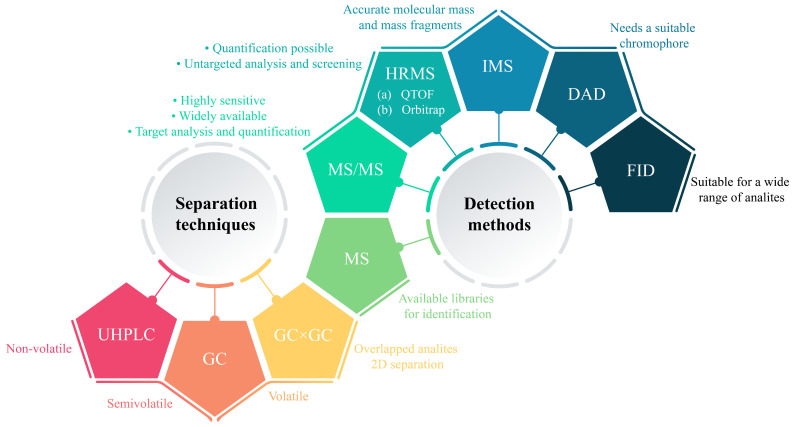
Common analytical techniques for the NTA of plastic FCMs.

The instrumental analysis of NIAS from plastic FCMs requires the integration of advanced separation and detection instruments to address the chemical diversity and complexity of migrants. In this sense, UHPLC and GC coupled with mass spectrometry (MS), MS/MS, and HRMS enable the precise identification and quantification of unknowns in non-targeted workflows [2]. A summary of the most used analytical techniques is depicted in Figure 2.

### 5.1. Separation Techniques

As evidenced by the chromatographic conditions reported across the reviewed studies, UHPLC dominates the analysis of non-volatile and semi-volatile NIAS, particularly for polar oligomers, polymer additives, and degradation products. Reversed-phase C18 columns (1.7–1.9 µm particle size) are the most prevalent, paired with H2O:MeOH or H2O:ACN gradients, usually with 0.1% formic acid or ammonium acetate (5–20 mM) added to enhance ESI efficiency. For instance, Aznar et al. [6] employed a C18 column (2.1 × 100 mm, 1.7 µm) with a 10 min gradient (H_2_O:MeOH, 0.1% formic acid) at 0.4 mL/min. Similarly, Cariou et al. [23] analyzed extracts from inner polyester coatings using a C18 column (2.1 × 100 mm, 1.9 µm) with a 20 min H_2_O:ACN gradient (10 mM ammonium acetate), demonstrating the method’s robustness for the separation of oligoesters. Shorter columns (e.g., 50 mm) and faster gradients (8–15 min) were adopted for high-throughput workflows, as seen in the study by Hu et al. [33], who characterized PA kitchenware migrants using an 8 min gradient (0.3 mL/min). Column chemistry can play a role in resolving specific NIAS classes. For instance, Gómez-Ramos et al. [30] employed a C8 column (2.1 × 100 mm, 1.8 µm) with a 20 min gradient (H_2_O:MeOH, ammonium formate/formic acid), identifying caprolactam, bis(2-methoxyethyl) adipate, additives, and cyclic oligomers in multilayer plastic packaging materials for fruit purée and juice by leveraging the column’s intermediate hydrophobicity. Similarly, in the work conducted by Tian et al. [56], PP and Tritan™ migrants were identified using a phenyl–hexyl column (3.0 × 100 mm, 2.7 µm) with a H_2_O:MeOH gradient, enabling also π-π interactions to enhance the separation of aromatic compounds.

The integration of ion mobility spectrometry (IMS) with UHPLC-QTOF-MS has increased the identification capabilities for co-eluting NIAS by incorporating collision cross-section (CCS) values as an additional analytical dimension. For instance, Ubeda et al. [60] conducted IMS to resolve cyclic and linear PLA oligomers, using CCS values to confirm structural assignments. Similarly, Vera et al. [64] employed UHPLC-IMS-QTOF-MS to identify antioxidant degradation products (e.g., Irganox 1010 and Irganox 1076 breakdown compounds) in PE films. In a later study, Vera et al. [66] extended this approach to EVA corks, demonstrating the versatility of IMS in improving identification confidence for structurally similar NIAS across diverse polymer matrices. However, IMS adds complexity to analytical workflows and increases instrument costs, which may limit its widespread adoption. Additionally, the limited availability of comprehensive CCS reference libraries for many NIAS currently constrains confident identification and requires further development to maximize IMS utility in routine NIAS screening.

On the other hand, GC remains indispensable for volatile and semi-volatile NIAS. Among different analytical setups, non-polar columns (e.g., DB-5MS and HP-5MS) with 5% phenyl–methyl polysiloxane stationary phases (30 m × 0.25 mm × 0.25 µm) and temperature gradients (40–320 °C at 5–20 °C/min) are standard [28,47]. Furthermore, Cincotta et al. [24] identified aliphatic aldehydes, unsaturated aldehydes, ketones, and hydrocarbons using a CP-Wax 52 CB column (60 m × 0.25 mm × 0.25 µm) with a 5 °C/min ramp to 240 °C, achieving the separation of structurally similar volatile compounds. Two-dimensional GC (GC × GC) also emerged as a useful strategy for complex mixtures, where conventional GC is not able to resolve co-eluting peaks. Hao et al. [32] coupled a DB-5MS primary column (30 m × 0.25 mm) with a DB-17MS secondary column (1 m × 0.25 mm) in a GC × GC-HRMS workflow, detecting 267 semi-volatile organic compounds (SVOCs) in rPET using orthogonal column selectivity. Similarly, Li et al. [39] coupled a semi-non-polar HP-5MS primary column (30 m × 0.25 mm) with a semi-polar DB-17MS secondary column (1 m × 0.25 mm), resulting in 1247 tentatively identified VOCs, including hydrocarbons, benzenoids, organic oxygen compounds, lipids, and lipid-like compounds, demonstrating the applicability of GC × GC in NTA.

The reviewed studies highlight the adaptability of LC and GC methods to diverse NIAS profiles, with UHPLC being the preferred technique for polar/non-volatile analytes and GC×GC being able to address the increased complexity of recycled materials. Column chemistry, gradient design, and temperature optimization remain important in balancing resolution, analysis time, and sensitivity, underscoring the need for method optimization. New separation dimensions, such as IMS, further enhance identification capabilities, driving the adoption of multidimensional and hyphenated techniques.

### 5.2. Detection Methods

The detection of NIAS in plastic FCMs relies on MS platforms tailored to the physicochemical properties of migrants, with HRMS and hybrid systems serving as the cornerstone for sensitivity, selectivity, and structural elucidation. The reviewed studies emphasize the critical role of mass analyzer selection, ionization modes, and acquisition strategies in addressing the complexity of NIAS, ranging from low-molecular-weight volatiles to high-molecular-weight oligomers.

HRMS systems, including QTOF and Orbitrap analyzers, dominate non-targeted screening due to their ability to deliver accurate mass measurements (<5 ppm exact mass error) and isotopic profiles for elemental formula assignments. For example, Aznar et al. [6] employed UHPLC-QTOF-MS in MS^E^ mode, an advanced DIA approach, which enables the simultaneous acquisition of MS/MS fragmentation spectra at low and high collision energies (CEs) during the same run. Similarly, Cariou et al. [23] utilized UHPLC-HRMS (Q-Orbitrap) to acquire one-dimensional full-scan (FS) data (120,000 FWHM) as well as targeted-MS^2^ (15,000 FWHM) for structural elucidation. Orbitrap systems are particularly suitable for high-resolution applications, standing out for resolving isobaric compounds, as demonstrated by Díaz-Galiano et al. [25], who operated HRMS using a combination of FS and data-dependent tandem mass spectrometry (ddMS^2^) modes. The FS mode employed a resolution of 120,000 FWHM with a scan range of 100–1000 *m*/*z*. Between every two FSs, 10 ddMS^2^ scans were acquired. The ddMS^2^ analysis trigger algorithm was based on minimum ion intensity (1 × 104) and a dynamic exclusion list. The ddMS^2^ scan employed a resolution of 15,000 FWHM with an isolation window of 1 *m*/*z* and a stepped CE mode in higher-energy collisional dissociation. While QTOF systems offer slightly lower resolving power, they are often preferred for faster acquisition speeds and higher throughput, which can be advantageous for large-scale screening workflows.

**Figure 3:**
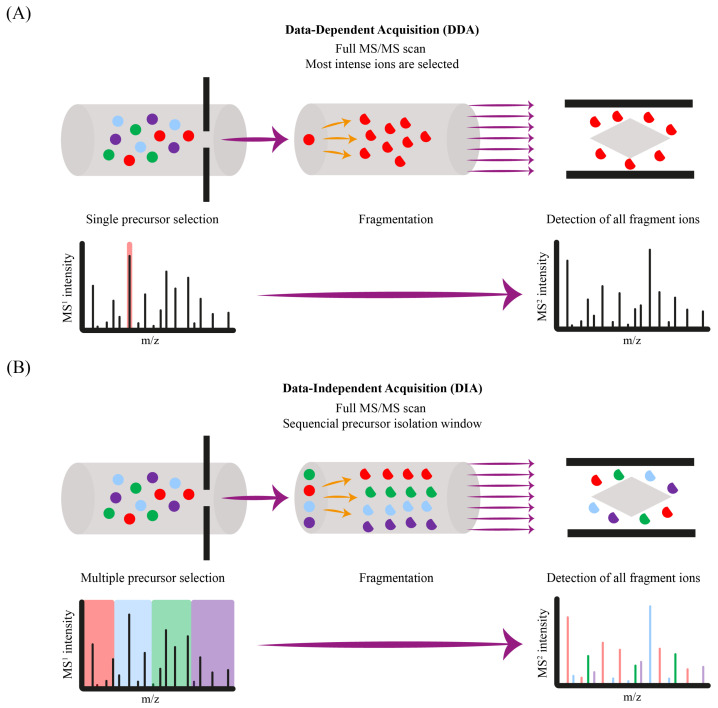
Schematic comparison between: (**A**) data-dependent acquisition (DDA) and (**B**) data-independent acquisition (DIA).

The high resolving power of these systems is particularly advantageous for polymer-derived NIAS, such as linear and cyclic oligomers. In this sense, non-targeted MS/MS acquisition allows for structural elucidation through precursor ion isolation and fragmentation, with both DIA and DDA modes (Figure 3). This improves identification confidence, particularly for isobaric or co-eluting compounds with unknown or overlapping fragmentation patterns.

In the work conducted by Bauer et al. [20], UHPLC-QTOF-MS was used, applying DIA-MS^2^ in sequential windowed acquisition of all theoretical MS (SWATH) mode. By applying SWATH acquisition, the full *m*/*z* range of precursor ions is continuously stepped along average mass windows of 20–85 Da, obtaining MS/MS data of much narrower precursor ion ranges and facilitating the recording of all detectable peaks in a sample with high-quality deconvoluted fragment mass spectra. Compared to DDA, which relies on intensity-based precursor selection and may miss low-abundance signals, DIA (including SWATH) ensures comprehensive MS/MS coverage but imposes higher data processing demands. This includes an increased risk of false positives or misannotations due to the complex spectral deconvolution required when many co-eluting compounds are fragmented simultaneously. As such, the choice between DIA and DDA depends on the sample complexity, target analyte profile, and available data analysis tools.

Ionization methods are pivotal in determining the scope of NIAS detection, with ESI, EI, and atmospheric pressure chemical ionization (APCI) serving distinct roles based on analyte polarity and volatility. These ionization techniques collectively address the chemical diversity of NIAS. In the first place, ESI, being predominant in LC-MS workflows, ionizes polar and high-molecular-weight compounds through protonation ([M + H]^+^) or deprotonation ([M − H]^−^), making it ideal for additives, oligomers, and acidic/basic compounds. For instance, the ESI+ mode is widely used to detect additives, like erucamide, oleamide, phthalates, or antioxidant degradation products, while ESI- excels for acidic species, such as palmitic acid or stearic acid [63,68]. The soft ionization nature of ESI preserves molecular ions, enabling HRMS characterization of labile compounds. In GC-MS, EI (70 eV) remains the standard, generating reproducible fragment-rich spectra for library matching (e.g., NIST). This technique is indispensable for volatile NIAS like aldehydes and hydrocarbons [24] or antioxidants and volatile additives [36]. However, the extensive fragmentation patterns of EI make the differentiation of isomers or structurally related substances difficult, where orthogonal separation (e.g., GC × GC) offers significant advantages, as has been previously mentioned [13]. Finally, APCI ionizes non-polar and thermally stable compounds via gas-phase reactions, reducing in-source fragmentation compared to EI. In GC workflows, APCI enhances the identification capabilities by preserving more precursor ions, as shown in the work conducted by Su et al. [54]. However, commercially available libraries for APCI fragmentation patterns are scarce, requiring significant efforts for structural elucidation.

While chromatographic techniques are predominant in the analysis of NIAS in plastic FCMs, a minority of studies employed alternative methods such as spectroscopy or elemental analysis. For example, Portesi et al. [49] investigated inorganic residues and contaminants in LDPE pellets and films using a multi-technique approach combining Raman spectroscopy, micro-Raman imaging, and inductively coupled plasma mass spectrometry (ICP-MS). The study highlighted the presence of unintended inorganic additives or contaminants, likely introduced during polymer processing, and emphasized the complementary role of Raman spectroscopy and ICP-MS in characterizing both organic and inorganic NIAS, complementing the broader reliance on LC-MS and GC-MS approaches.

## 6. Data Processing and Analysis

### 6.1. Compound Identification Workflows

**Figure 4:**
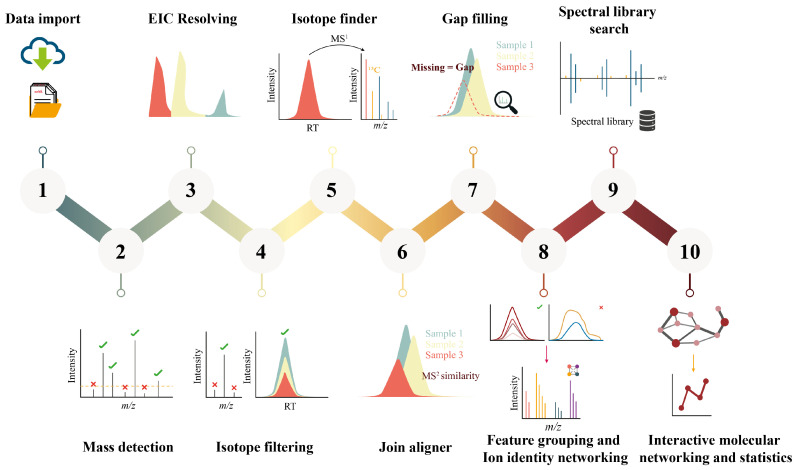
Common steps in data processing workflows for compound identification.

The non-targeted analysis of NIAS from plastic FCMs generates vast and complex datasets, requiring robust data processing workflows and advanced identification strategies. These workflows typically involve peak detection, feature alignment, spectral deconvolution, database matching, and confirmation or quantification with analytical standards, if available. However, the high dimensionality of the data often results in challenges such as feature redundancy, false positives, and difficulties distinguishing relevant signals from background noise. Additionally, reproducibility can be compromised by differences in data processing software, particularly when using proprietary algorithms, which may affect feature detection and compound annotation outcomes. The most common data processing steps for compound identification [70] are summarized in Figure 4.

Software platforms such as MassLynx and UNIFI (Waters Corporation, Milford, MA, USA), Compound Discoverer (Thermo Fisher Scientific, Waltham, MA, USA), and MS-DIAL [71] enable automated or semi-automated data processing and tentative compound identification. For instance, Aznar et al. [6] manually processed UHPLC-QTOF-MS data using the MassLynx software and the ChemSpider [72] and SciFinder (CAS, Columbus, OH, USA) databases, identifying 37 plasticizers and oligomers via exact mass and fragment ion matching, while Bauer et al. [20] employed the SciexOS software (SCIEX, Framingham, MA, USA) for automated peak grouping and deconvolution, cross-referencing spectra against an in-house library of 237 IAS/NIAS. In that work, the identification criteria were adopted from the SANTE 11813/2017 guidelines [73], including a mass accuracy of ±5 ppm, a retention time shift of ±0.1 min, two diagnostic ions (precursor and fragment ions) with an ion ratio tolerance of ±30%, and a <20% difference between the observed and the theoretical isotope distribution. Similarly, Gómez-Ramos et al. [30] combined automatic feature processing and elemental composition assignment with the SciexOS software for the manual tentative identification of detected compounds using the ChemSpider and MS/MS spectral databases, such as METLIN [74] or MassBank [75], in addition to in silico fragmentation tools and searches in the scientific literature. In that case, a mass tolerance of ±10 ppm, an isotopic pattern distribution, factors for ring and double bonds, and MS/MS spectrum were considered. Although these identification approaches focus on spectral quality and database annotation, recent workflows increasingly incorporate prioritization strategies based on toxicological relevance, such as risk-based filtering or Threshold of Toxicological Concern (TTC) concepts. While these aspects are addressed in detail in Section 7, they are crucial to reduce the analytical burden and focus follow-up efforts on compounds with higher safety concerns.

Spectral libraries, such as MassBank, the NIST/EPA/NIH Mass Spectral Library, and the NIST GC Retention Index (RI) Database (National Institute of Standards and Technology), as well as in silico fragmentation tools, such as CFM-ID [76], are critical for structural elucidation and tentative identification. For example, Chen et al. [18] pre-processed GC-MS data (including peak detection, alignment, blank subtraction, and identification) with MS-DIAL using RI calculations based on n-alkane (C9–C36) as well as NIST library matching (score ≥75%, RI tolerance ±20 when available). In their work, a total of 80 compounds were detected in PE and rPE, and 70 of them were tentatively identified, including hydrocarbons, esters, aldehydes, alcohols, ethers, acids, benzene derivatives, ketones, amides, and piperazine derivatives. Nevertheless, library gaps for the identification of NIAS remain a persistent challenge. For instance, Miralles et al. [42] processed GC-Q-Orbitrap HRMS data using the Compound Discoverer software coupled to the NIST library, detecting 374 features in rLDPE extracts. However, only 83 substances were tentatively identified (score ≥ 90%, RI tolerance ± 50 when available), including plasticizers, surfactants, stabilizers, emulsifiers, LDPE oligomers, and other NIAS with industrial applications. That means that the other 291 detected features could not be tentatively identified with a sufficient level of confidence using automated procedures, and they would require significant efforts in manual annotation via fragment interpretation and literature cross-referencing. In such cases, RI prediction models can help narrow down plausible candidates, while in silico fragmentation tools provide ranked fragment matches against proposed structures, increasing confidence in annotation even when spectral libraries lack reference spectra.

In addition to in silico fragmentation, molecular networking (MN) tools, such as GNPS [77] and Cytoscape [78], are bridging library gaps. MN is a computational approach designed to facilitate chemical identification in non-targeted studies using HRMS and MS/MS data by clustering MS/MS spectra based on spectral similarities [79]. Beyond enhancing data visualization, MN offers the advantage of being constructed directly from the same UHPLC-HRMS datasets used in conventional analyses, integrating chromatographic parameters (e.g., retention time), detection features (e.g., exact mass and adducts), and quantitative data. Feature annotation within MN is achieved by comparing measured spectra against spectral databases, generating tentative identifications weighted by similarity scores. However, the reliability of tentative identifications may be limited by variability in ionization efficiencies and unknown response factors, particularly for polar, reactive, or thermally labile NIAS. In the work conducted by Omer et al. [46], UHPLC-Q-Orbitrap HRMS and MN were used to investigate and classify oligomers in bioplastic packaging materials. The acquired data were manually processed for peak integration, molecular mass determination, and elemental formula generation, followed by spectral comparison with databases. To refine feature detection, the Progenesis QI software v. 2.4.6911.27652 (Nonlinear Dynamics, Durham, UK) was used for alignment, peak picking, adduct deconvolution, feature filtration based on strict acceptance criteria, and the extraction of MS/MS spectra. Then, MN was implemented in Python 3.7 and visualized in Cytoscape v.3.7.1, obtaining clusters of structurally related compounds for tentative identification. The global MN contained 596 nodes, with 491 spectral library hits and 176 putative proposals matching the exact masses measured by HRMS. The manual processing revealed 152 features, out of which 96 compounds were identified and grouped into three classes of oligomers: PLA, polybutylene adipate terephthalate (PBAT), and polybutylene succinate (PBS). However, MN revealed a new series of azelaic acid (AZA) oligomers, which remained unidentified using the manual method. Therefore, the study carried out by Omer et al. [46] demonstrates the potential of MN as an emerging technology that can be readily implemented in the packaging field, improving data visualization and enabling the classification of unknown migrating substances.

The integration of multi-platform data is also becoming essential for comprehensive NIAS profiling. For example, Tisler et al. [58] combined UHPLC-QTOF-MS data processing (MS-DIAL and others) with quantitative non-target analysis (qNTA) by quantitative structure-property relationship (QSPR) modeling to predict the response factor (peak intensity divided by concentration) for each compound using molecular descriptors. Estimated concentrations were obtained by dividing the predicted response factor by the total peak intensity, accounting for isotopes, adducts, and known in-source fragments and predicting the migration behavior of NIAS in reusable plastic bottles.

### 6.2. Identification Confidence and Confirmation

**Figure 5:**
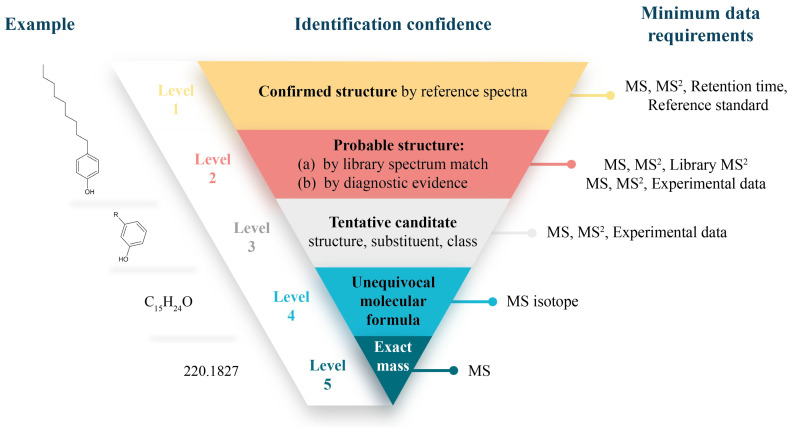
Summary of identification confidence levels and identification criteria. Adapted with permission from Schymanski et al. [80], 2014, American Chemical Society.

Confidence in NIAS identification can be systematically categorized using the framework introduced by Schymanski et al. [80], which ranks confidence from Level 1 (confirmed structure with a reference standard) to Level 5 (exact mass). The described confidence levels and their identification criteria are summarized in Figure 5. This tiered approach underscores the need for transparent reporting, as most NIAS identifications fall into Levels 2–4 due to unavailable analytical standards and limited spectral data in libraries and databases. However, communication regarding identification confidence remains inconsistent across studies, highlighting the need for harmonized reporting standards in NTA.

The confirmation and quantification of NIAS in plastic FCMs depend on the availability of analytical standards, which are essential for achieving high confidence in identification (Level 1) and accurate quantification. For instance, García-Ibarra et al. [28] confirmed 17 out of >40 compounds (approximately 42%) based on retention time and their respective spectral data with analytical standards, including n-alkanes, toluene diisocyanates, BHT, benzophenone, and various phthalate esters [28]. Similarly, Yusà et al. [68] confirmed the identity of 12 out of 24 tentatively identified substances (50%) using standards. The remaining detected peaks could not be confirmed due to the lack of commercial standards, and they were considered as tentatively identified. These figures illustrate the practical limitations of full confirmation, with a significant proportion of NIAS remaining at lower confidence levels due to lack of commercially available reference materials. In this sense, the scarcity of analytical standards for many NIAS, particularly oligomers and degradation products, necessitates innovative workarounds. Ubeda et al. [60] faced this challenge by semi-quantifying PLA oligomers using a cyclic ester oligomer formed by adipic acid (AA), diethylene glycol (DEG), and isophtalic acid (IPA), with AA-DEG-IPA-DEG as the standard.

In the work conducted by Vera et al. [64], analytical standards of the proposed compounds were analyzed under the same conditions as the samples to confirm their identity (retention time, CCS values, and fragment matches), including a CCS deviation of ≤2% as the confirmation criterion. To enable quantification, calibration curves were constructed using a dilution series of the standards prepared in EtOH. When the standard was not commercially available, the compound was quantified using another standard with a similar molecular structure. This approach, while pragmatic, introduces uncertainties in concentration estimates, especially when the analytes and the compounds used as standards exhibit divergent physicochemical properties. In this regard, quantification challenges are exacerbated by matrix effects, which may alter ionization efficiency and the detector response. For the purpose of correcting matrix effects, Cincotta et al. [24] quantified volatile NIAS in PET bottled mineral water using standard addition calibration. They employed standards of nonanal, tetradecanal, (E)-oct-2-enal, nonadecane, limonene, toluene, and dibutyl phthalate (DBP). In addition to these substances, nonanal was used to calibrate the quantification of aliphatic aldehydes ranging from C8 to C11, while tetradecanal served for those from C12 to C16. Unsaturated aldehydes and ketones were quantified using the (E)-oct-2-enal calibration curve, whereas nonadecane was applied for the quantification of aliphatic hydrocarbons.

In general, the lack of NIAS reference standards is a major bottleneck for their identification, quantification, and risk assessment. Furthermore, the synthesis of custom standards, though ideal, is often cost-prohibitive and time-consuming. To overcome the lack of reference standards, Cariou et al. [23] proposed the organic synthesis of linear and cyclic oligomers derived from neopentyl glycol and IPA units, both in their native and deuterated forms. To achieve this, they employed a stepwise synthesis approach, carefully controlling cyclization through a challenging macrolactonization step as the final stage [81]. This strategy ensured the production of pure analytical batches while optimizing recovery yields and minimizing the waste of costly labeled precursors.

The uncertainties introduced by surrogate-based quantification and matrix effects have direct implications for risk assessments. For instance, semi-quantitative estimates may underestimate or overestimate exposure levels. Standardized protocols for standards selection, such as prioritizing structurally homologous compounds or applying ionization efficiency corrections, could mitigate these errors. To improve comparability and transparency, minimum quality reporting standards should be followed, such as clearly indicating the confidence level of identification (e.g., Schymanski levels) and specifying whether quantification is based on reference or surrogate standards. In summary, while analytical standards are indispensable for precise NIAS confirmation and quantification, their limited availability drives reliance on semi-quantitative methods, introducing uncertainties that complicate risk assessments. Future advancements in standard availability, coupled with harmonized protocols for surrogate selection and matrix effect compensation, are critical to enhancing the accuracy and reliability of NIAS characterization in plastic FCMs.

## 7. Risk Assessment

The risk assessment of NIAS in plastic FCMs requires evaluating both exposure levels obtained from the quantification (or semi-quantification) and assessment of toxicological hazards. Most of the reviewed studies followed established guidance frameworks, such as the TTC approach based on the Cramer classification, which sets exposure thresholds according to the chemical structure [82]. They also compared findings with regulatory limits, including the SML defined in Regulation (EU) No 10/2011 [7]. However, these frameworks present some limitations. The TTC approach, while useful for prioritization, may not account for the combined effects of multiple co-occurring substances (mixture toxicity), and the Cramer classification tends to oversimplify toxicity by focusing on structural features without considering functional activity or metabolic pathways. Moreover, the TTC model is not applicable to substances with potential genotoxicity or to poorly characterized NIAS lacking adequate structural information. The use of SMLs is also constrained, as most NIAS lack specific migration limits due to absent toxicological data.

### 7.1. Estimated Daily Intake and Tolerable Daily Intake

The exposure assessment from FCMs is typically derived from migration studies using standardized food simulants under controlled conditions (as seen in Section 4.1). The migration values obtained (usually in mg/kg of the food simulant) are then translated into human exposure scenarios as the estimated daily intake (EDI) by applying conventional assumptions. These include a food packaging surface-to-food mass ratio of 6 dm^2^/kg, a daily intake of 1 kg of packaged food per person, a consumption factor (CF) representing the fraction of the daily diet for a specific material, and a default body weight (bw), which is commonly 60 kg for adults in European assessments [83]. However, these default values may not adequately reflect sensitive or high-exposure populations, such as infants, toddlers, or pregnant women. For instance, infants have higher food consumption relative to their body weight, potentially leading to proportionally greater exposure, especially in scenarios involving baby food packaging [7,83]. Accordingly, the EDI is calculated using the following expression:

EDI (mg/kg bw/day) = [Migration (mg/kg food) × Food intake (1 kg/day) × CF]/bw(1)

Once the EDI of a substance migrating from FCMs is calculated, it is compared with available toxicological reference values, most commonly the tolerable daily intake (TDI), which represents the amount of a substance that can be ingested daily over a lifetime without appreciable health risk. TDIs are often established by authorities, such as the European Food Safety Authority (EFSA), the U.S. Environmental Protection Agency (EPA), or the World Health Organization (WHO), based on toxicological studies and the application of safety factors to account for uncertainties [84]. In cases where a TDI is available, the hazard quotient (HQ) is used as an indicator to assess risk. The HQ is calculated as the ratio between the estimated exposure and the TDI as described in the follow equation:

HQ = EDI/TDI(2)

An HQ value of <1 suggests that exposure is within the acceptable safety margins and is unlikely to pose a risk, whereas an HQ value of ≥1 may indicate potential concern and warrants further investigation. For example, if an EDI of 0.002 mg/kg bw/day is estimated for a substance with a TDI of 0.01 mg/kg bw/day, the HQ would be 0.2, implying no immediate risk under current exposure assumptions.

In the work conducted by Kim et al. [34] the TDI values for melamine and formaldehyde from EFSA [85,86] were applied to evaluate the risk associated with the migration of these substances from melamine–formaldehyde utensils after UV irradiation, using consumption and food-type distribution factors based on the U.S. Food and Drug Administration (FDA) guidelines [87]. As only one food simulant was used, the food-type distribution factor was set to one. EDI values were compared to the TDI to assess potential health risks. Results indicated that melamine exposure (EDIs) ranged from 0.14% to 1.36% of the TDI in the control conditions and 0.26 to 11.78% under UV exposure. For formaldehyde, exposure ranged from 1.99 to 3.95% in the control samples and 2.23 to 7.95% under UV exposure. These values demonstrate that the migration levels of both compounds remain well below the respective TDI thresholds, indicating a low health risk for consumers under the tested conditions. However, these results did not account for the statistical uncertainty associated with EDI estimates, such as confidence intervals or variability in migration measurements, which may impact the interpretation of risk, especially near regulatory thresholds.

Limitations exist, particularly when TDIs are lacking, as is common for many NIAS, or when data are insufficient for full hazard characterization. In such cases, alternative approaches like the TTC are applied. Additionally, HQ-based assessments typically consider substances individually and do not account for cumulative or mixture effects, which may underestimate the true risk posed by complex NIAS profiles in plastic FCMs. The U.S. FDA [88] recommends the use of additive models, such as dose addition, for mixture risk assessments when components have similar modes of action, although this approach is not yet routinely applied in NIAS evaluations.

### 7.2. Threshold of Toxicological Concern Approach

The Cramer decision tree, developed by Cramer et al. [89] and later refined by EFSA [90], is a systematic approach for classifying chemical substances into one of three toxicity classes (Class I, II, or III) based on their structural features and potential toxicological concerns. This classification system evaluates molecular structure through a series of 33 questions that assess the presence of functional groups, structural alerts, and metabolic activation potential that may indicate toxicity. Class I (low toxicity) includes substances with simple chemical structures and efficient metabolic pathways (e.g., sugars and amino acids) that suggest low toxicity potential, assigned a TTC threshold (TDI values) of 30 µg/kg bw/day. Class II (intermediate toxicity) contains moderately complex structures (e.g., esters and alcohols) with some reactive groups but no significant toxicity alerts, assigned a threshold of 9 µg/kg bw/day. Class III (high toxicity) comprises substances with structural features suggesting potential toxicity (e.g., aromatic amines, conjugated double bonds, and heterocyclic compounds) or those that cannot be confidently assigned to Classes I or II, assigned the most conservative threshold of 1.5 µg/kg bw/day.

This system has been widely adopted in NIAS risk assessments. For instance, Bauer et al. [20] reported that several NIAS, such as ε-caprolactam oligomers, bis(2-hydroxyethyl) terephthalate (BHET), AA-DEG, and cyclic PES oligomers, were found in baby food at concentrations exceeding the 0.01 mg/kg migration limit set for NIAS by Regulation (EU) No 10/2011 [7]. In particular, ε-caprolactam was detected in 67% of baby food samples (0.01–7.50 mg/kg), remaining below the SML of 15 mg/kg. However, its cyclic oligomers, BHET, and AA-DEG often exceeded the 0.01 mg/kg NIAS threshold. Other semi-quantified oligomers reached concentrations of up to 4 mg/kg in some samples, suggesting that consumer exposure was higher. To assess toxicological relevance, compounds were categorized according to the Cramer decision tree using the Toxtree software [91]. Cyclic oligomers were mainly classified as Cramer Class III, while linear oligomers were classified as Class II. For an average infant weighing 10 kg, this equates to a tolerable intake of 0.015 mg/day of Class III oligomers. However, the calculated intake for AA-DEG from a single baby food pouch exceeded this limit by over 30-fold, indicating a potential safety concern when applying the TTC concept to these substances. It should be noted that the TTC thresholds incorporate a 100-fold uncertainty factor to account for inter- and intra-species variability [90], yet such exceedances may still warrant further toxicological investigation, particularly in sensitive subpopulations.

However, the TTC approach has limitations, particularly for genotoxic or bioaccumulative substances. In the case of potential genotoxic substances, Commission Regulation (EU) No 10/2011 sets a stricter migration limit of 0.00015 mg/kg. In the study by [42], 9 substances exceeded the general 0.01 mg/kg limit, while 45 exceeded 0.00015 mg/kg. Some compounds were IAS and complied with their SMLs, indicating no immediate risk from an rLDPE film. However, 291 unidentified substances were also detected, for which toxicity and genotoxic potential remain unknown. The authors recommended further studies to identify these compounds and assess their individual and mixture toxicity, ideally using bioassays to support comprehensive risk assessments. For such high-risk NIAS, the EFSA recommends a default threshold of 0.0025 µg/kg bw/day [90]. In practical terms, these low thresholds pose significant analytical challenges, as the corresponding migration levels are often close to or below the limits of detection usually achievable with current NTA methods. As a result, screening workflows may fail to detect relevant compounds or require highly sensitive targeted follow-up analyses to confirm potential genotoxicants.

### 7.3. Bioassays

In vitro bioassays are increasingly integrated into hazard characterization to address data gaps in NIAS risk assessments. For example, Mallen et al. [40] prioritized 16 NIAS found in epoxy coatings and evaluated their mutagenic and genotoxic potential. They synthesized an oligomer based on tetramethyl bisphenol F-based diglycidyl ether (TMBPF-DGE) resin and hydroquinone and tested it using two in vitro assays: a bacterial reverse mutation test (Ames test) and an in vitro micronucleus test with human lymphocytes. The Ames test showed no mutagenic activity, and the micronucleus test revealed no genotoxicity. These results suggested that the TMBPF-DGE+HQ oligomer did not pose a genotoxic or mutagenic risk, warranting further in vivo evaluations. Additionally, the study also assessed the overall migration of substances from the V70 coating using a modified Ames test, finding no mutagenicity in the migration products. The authors acknowledged the limitations of these bioassays, including their focus on a limited number of toxicological endpoints, challenges in testing complex mixtures, the lack of full validation for some assays, and the inability of in vitro assays to fully replicate in vivo conditions, as well as concerns regarding sensitivity and specificity, which may lead to false negatives or missed endpoints relevant to long-term exposure. Nevertheless, bioassays remain a valuable tool to identify potential hazards, prioritize substances for further testing, and complement chemical analysis in the safety evaluation of FCMs.

These methods complement the TTC approach by providing mechanistic insights; however, extrapolating in vitro responses to actual human health risks remains a significant challenge. Overall, the risk assessment of NIAS remains a multifaceted challenge that requires balancing conservative default approaches like TTC with emerging toxicological evidence. While tools such as the Cramer classification and SMLs provide useful regulatory benchmarks, they have limitations, particularly in addressing genotoxicity, cumulative exposures, and the unique hazards posed by recycled and bio-based materials. Advancing NIAS risk assessments will require the development of dedicated toxicity databases, the integration of in vitro and in silico tools, and the implementation of probabilistic models to better capture exposure variability. Coordinated efforts between regulatory bodies, industry stakeholders, and academic researchers will be essential to bridging existing gaps and safeguarding public health in the context of increasingly complex FCM compositions.

## 8. Conclusions and Future Perspectives

The analysis of NIAS in plastic FCMs has advanced significantly in recent years, driven by the growing recognition of their potential health risks and the regulatory push for safer, more sustainable packaging. While conventional polymers like PET, PE, and PP remain focal points due to their ubiquity, emerging materials such as bioplastics, recycled polymers, and composite systems introduce new dimensions of uncertainty, particularly regarding novel migrants and legacy contaminants. It is important to note that some issues discussed herein, such as mixture toxicity modeling or quantitative uncertainty analysis, are not explored in depth, since they were not covered extensively by the reviewed literature.

Regarding sample preparation, the reviewed studies underscore that no single extraction method is sufficient for comprehensive NIAS profiling, given the chemical diversity and complexity of plastic FCMs. A concerted effort toward harmonized protocols, interlaboratory validation, and tiered extraction schemes tailored to polymer type and intended use, coordinated by international bodies such as EFSA, is needed. Future efforts must also prioritize greener, safer solvent systems and the development of advanced analytical workflows to address the evolving challenges posed by novel materials, recycling-related contaminants, and regulatory demands.

Advances in analytical instrumentation, notably UHPLC, GC, and GC×GC coupled to HRMS using Orbitrap or QTOF-MS analyzers, and more recently IMS, have revolutionized non-targeted screening, enabling the detection and identification of thousands of previously uncharacterized NIAS. However, the field remains challenged by critical gaps in spectral libraries, reference standards, and harmonized protocols, leading to overreliance on tentative identifications and semi-quantitative estimates. To address this, initiatives like open-access databases should be expanded to include polymer-specific NIAS spectra, fragmentation patterns, and CCS values. Clear minimum reporting standards, covering identification confidence, compound-specific response factors, and data transparency, should be established to improve reproducibility and comparability. The widespread adoption of the TTC approach and the Cramer classification for risk assessments reflects a pragmatic approach to prioritizing hazards, yet these frameworks struggle to address genotoxicity, bioaccumulation, and mixture effects, issues increasingly relevant to recycled and bio-based materials.

Several priorities are essential to advancing NIAS research and regulation, such as increased collaborative efforts to expand open-access databases with polymer-specific NIAS spectra, fragmentation patterns, and CCS values. Moreover, synthesizing reference standards for high-priority migrants, particularly oligomers and degradation products, is critical to improving quantification accuracy. Combining non-targeted HRMS with in vitro bioassays (e.g., mutagenicity and endocrine disruption assays) and computational toxicology tools will enable mechanistic insights into NIAS hazards, bridging the gap between detection and risk characterization. Current risk assessments largely evaluate NIAS in isolation, underestimating the potential synergistic or additive effects of chemical mixtures. Operationally, this calls for implementing mixture toxicity models (e.g., dose addition) and defining default mixture assessment factors where data are lacking.

Recycled and bio-based polymers necessitate tailored migration testing and risk assessments that account for contaminants introduced during recycling, biodegradation byproducts, and interactions between natural and synthetic matrices in composites. The shift toward circular economies and bio-based alternatives must be accompanied by rigorous safety-by-design principles. For instance, biodegradable polymers should be engineered to minimize toxic degradation products, while recycling processes must incorporate advanced decontamination steps to eliminate legacy substances.

Finally, existing regulations, such as Regulation (EU) No 10/2011, focus on intentional additives and predefined SMLs, leaving NIAS oversight fragmented. Harmonized guidelines mandating non-targeted screening for novel materials, coupled with transparency in reporting unidentified peaks, will enhance consumer protection. Regulatory acceptance of non-targeted data, including provision for semi-quantitative thresholds and bioassay evidence, must be fostered through stakeholder engagement and scientific consensus.

In conclusion, the intersection of analytical chemistry, toxicology, and materials science will define the next era of NIAS research. Emerging technologies, such as IMS, machine learning, or artificial intelligence-driven spectral prediction, promise to deepen our understanding of NIAS migration, transformation, and biological impact. However, translating these advancements into actionable policies requires sustained collaboration among academia, industry, and regulators. Moving forward, operational roadmaps should prioritize the harmonization of analytical workflows, the coordinated generation of reference standards, the integration of mixture toxicity into risk models, and regulatory frameworks that incorporate non-targeted evidence. Ultimately, ensuring the safety of plastic FCMs demands a proactive, precautionary approach that balances innovation with rigorous hazard characterization, fostering public trust in sustainable packaging solutions while safeguarding global health.

## Figures and Tables

**Table 1:** Detailed summary of the 54 reviewed studies on NTA of NIAS in plastic FCMs.

FCMs (Polymers)	Sample Preparation	Analytical Technique	Instrumental Conditions	Mass Spectrometry	Data Processing	Identified Compounds	Risk Assessment	Ref.
Printed films (PP) and UV varnishes	(1) Extraction: EtOH 95%, room temp, 1 h (2) Migration: D2 (EtOH 95%), 60 °C, 10 days.	(1) GC-MS (2) UHPLC-IMS-QTOF-MS	(1) HP5-MS (30 m × 0.25 mm, 0.25 µm); 40 °C (2 min), 10 °C/min to 300 °C (2 min); 1 µL SL, 1 mL/min. (2) UPLC™ BEH C18 (2.1 × 100 mm, 1.7 µm), 0.3 mL/min, 5 µL inj., 35 °C, H_2_O:MeOH (0.1% formic), gradient, 13 min	(1) EI, FS (2) ESI(+/−), MS^E^	Manual: Chemdraw, MassFragment, NIST MS, Chemspider, and MassLynx. Analytical standards or technical mixtures	(**20**) Photoinitiators for UV curing, ester monomers, and reaction products (20 compounds)	TTC (Cramer) and ToxTree	[5]
Pellets and films (PLA and biodegradable fossil-based PES)	(1) Total dissolution/precipitation: DCM/EtOH. (2) Migration: A, B, D2 (EtOH 95%), 60 °C, 10 days	UHPLC-QTOF-MS	UPLC BEH C18 (2.1 × 100 mm, 1.7 µm), 0.4 mL/min, 10 µL inj., 40 °C, H_2_O:MeOH (0.1% formic), gradient, 10 min	ESI(+), MS^E^	Automatic: MassLynx, Chemspider, SciFinder, and MassFragment.	(**37**) AA, PA, BD cyclic oligomers, and PLA oligomers	-	[6]
Food storage containers (PP)	Migration: H_2_O, A, B, D2 (isooctane), 40 °C, 10 days, rotary evaporator and reconstitution (ACE)	GC × GC-HRMS (TOF)	Column 1: 8% phenyl (equiv.) polycarborane siloxane, 30 m × 0.25 mm, 0.25 µm; 45 °C (2.5 min), 20 °C/min 190 °C, 3 °C/min 300 °C (30 min). Column 2: 50% phenyl 50% methyl-polysilphenylene siloxane, 1.7 m × 0.1 mm, 0.1 µm; 75 °C (2.5 min), 20 °C/min 210 °C, 3 °C/min 300 °C (30 min); 1 µL SL, He at 30 psi	EI, 70 eV, FS 75–700 *m*/*z*	Automatic: ChromaToF, libraries, and the scientific literature. Confirmation with standards.	(**107**) Antioxidants, degradation products, contaminants, and 23 unknowns	-	[13]
(PE and rPE)	Migration: D2 (EtOH 95%), 60 °C, 10 days	GC-MS	HP-5MS (30 m × 0.25 mm, 0.25 µm), 1 µL SL 250 °C, He 1 mL/min; 50 °C, 10 °C/min 200 °C, 5 °C/min 250 °C (10 min), 5 °C/min 300 °C (6 min)	EI, 70 eV, 230 °C, FS 50–550 *m*/*z*	Automatic: MS-DIAL and NIST MS library. Multivariate analysis: SIMCA and SPSS.	(**80**) 10 hydrocarbons, 29 esters, 3 aldehydes, 9 alcohols, 2 ethers, 4 acids, 4 benzene derivatives, 4 ketones, 3 amides, 2 piperazine derivatives, and 10 unknowns	TTC (Cramer)	[18]
Baby food squeezes (PET and PE)	Migration: B, C, 40 °C, 10 days	UHPLC-QTOF-MS	Zorbax Eclipse Plus C8 (2.1 × 100 mm, 1.8 µm), 0.3 mL/min, 5 µL inj., 35 °C, H_2_O:MeOH (ammonium formate/formic), gradient, 20 min	ESI(+), DIA-MS^2^ using SWATH	Automatic: SciexOS, the literature, in silico MS^2^, and in-house databases (237 IAS/NIAS). Analytical standards.	(**42**) 3 IAS, 35 polyester oligomers (29 cyclic and 6 linear), and 4 NIAS (plasticizers)	TTC (Cramer)	[20]
Pellets and slotted spoon (PBT)	(1) Extraction: DCM, ACN, DMSO, EtOH 20%, 60 °C, 1 h, evaporation and reconstitution (2) Total dissolution/precipitation: DCM/2-propanol (3) Migration: H_2_O, 100 °C, 2 h	HPLC-DAD/MS	Multospher^®^ 120 RP C18 (3 × 250 mm, 5 µm), 0.4 mL/min, 10 µL inj., 60 °C, H_2_O (0.015% formic): 2-propanol, gradient, >85 min	ESI(+/−), FS	Manual processing and identification. Quantification with BHET.	(**27**) cyclic and linear oligomers	TTC (Cramer)	[21]
Pellets and bottles (PET, PETG, and Tritan)	(1) Extraction: DCM, ACN, DMSO, EtOH 20%, 60 °C, 1 h, evaporation and reconstitution. (2) Total dissolution/precipitation: DCM/2-propanol	HPLC-DAD/MS	Multospher^®^ 120 RP C18 (3 × 250 mm, 5 µm), 0.4 mL/min, 10 µL inj., 60 °C, H_2_O (0.015% formic): 2-propanol, gradient, >85 min	ESI(+/−), FS	Manual processing and identification. Quantification with BHET.	(**100**) linear and cyclic oligomers	TTC (Cramer) and ToxTree	[22]
Inner coatings from metallic cans (PES)	Extraction: 2 cm^2^ coating with 2 mL CAN, 40 °C, 24 h	UHPLC-HRMS (Q-Orbitrap)	Hypersil Gold C18 (2.1 × 100 mm, 1.9 µm), 0.4 mL/min, 10 µL inj., 40 °C, H_2_O:ACN (both 10 mM ammonium acetate), gradient, 20 min	H-ESI(+), FS, targeted-MS^2^	Automatic: The R environment, in-house databases, and the scientific literature. Response factors of internal standards.	(**125**) 84 oligoesters, epoxidized soybean oil, BADGE, benzoguanamine derivatives, and phenol-formaldehyde oligomers	-	[23]
Bottled mineral water (PET)	Extraction: HS-SPME DVB/CAR/PDMS fiber, 60 °C, 15 min, constant stirring	GC-MS (IT)	Desorption: 3 min, 260 °C SL, CP-Wax 52 CB (60 m × 0.25 mm, 0.25 µm); 60 °C (5 min), 5 °C/min 240 °C (20 min); He 10 psi	EI, FS 30–300 *m*/*z*	Manual: NIST library, RI, and the literature data. Aldehydes standards and standard addition calibration.	(**26**) 8 aldehydes (C8-C15), 9 unsaturated aldehydes (C7-C11), 5 hydrocarbons (C11-C19), 1 ketone, 1 terpene, 1 phthalate, and 1 aromatic hydrocarbon	-	[24]
Microwavable food containers (PET, PP)	Migration: A, 40 °C, 10 days	UHPLC-HRMS (Q-Orbitrap)	Accucore^TM^ C8 (2.1 × 100 mm, 2.6 µm), 0.35 mL/min, 30 °C, H_2_O:MeOH (ammonium formate/formic), gradient, 17 min	H-ESI(+), FS 100–1000 *m*/*z*/ddMS^2^	Automatic: Compound Discoverer, databases. MassFrontier. Analytical standards	(**16**) 1 maltose derivative of photoinitiator HMPP and 15 PPGs	-	[25]
Baby feeding bottles (PPSU)	(1) Total dissolution/precipitation. (2) Migration: D1, 70 °C, 2 h	Multitechnique (1H-NMR, SEC-DAD, HPLC-DAD/MS/AD/FLD, and GC-MS)	-	ESI(+), EI	Confirmation and quantification with reference standards.	Monomers, linear and cyclic oligomers and derivatives, and volatile and non-volatile migrants.	SML (R. 10/2011) TTC (Cramer) and ToxTree	[26]
Plastic candy wrappers	Extraction: ACN, 70 °C, 6 h, evaporation and reconstitution	GC-MS	ZB-5MS (30 m × 0.25 mm, 0.25 µm), 1 µL SL 300 °C, He (1 mL/min); 40 °C (2 min), 9 °C/min 300 °C (3 min)	EI,70 eV FS 35–500 *m*/*z*	Manual: NIST MS and Wiley libraries. Reference standards.	(**23**) n-alkanes (C21-C29), BHT, degradation products, plasticizers	TTC (Cramer), ToxTree	[27]
Corn and potato snacks, cookies, cakes packaging (PP)	Extraction: ACN, 70 °C, 24 h, and evaporation	GC-MS	ZB-5MS (30 m × 0.25 mm, 0.25 µm), 1 µL split 1:10 300 °C, He (1 mL/min); 120 °C (2.5 min), 9 °C/min 200 °C (2 min), 9 °C/min 300 °C (1 min), 20 °C/min 320 °C (7 min)	EI 70 eV, FS 40–400 *m*/*z*	Manual: NIST MS, Wiley libraries. Reference standards.	(>40) alkanes, aldehydes, alcohols, phthalates, citrates, adipates, phosphates, phenolic compounds, diisocyanates, and fatty acids.	SML (R. 10/2011) TTC (Cramer) and ToxTree	[28]
Films (PE, PET, PA, PP, and EVA)	(1) P&T: He 40 mL/min, 60 °C, 20 min (Vocarb 3000 Trap, Supelco, Bellefonte, PA, USA) (2) Extraction: ACN, 70 °C, 24 h. (3) Migration: E, 60 °C, 10 days, desorption with ACE; D2 (isooctane), 20 °C, 2 days	(1) P&T-GC-MS (2) GC-MS	(1) P&T: ZB-624 (30 m × 0.25 mm, 1.4 µm); 35 °C (4 min), 5 °C/min 210 °C (5 min); He 1 mL/min. (2) ZB-5MS (30 m × 0.25 mm, 0.25 µm), 1 µL SL 300 °C, He (1 mL/min); 50 °C (3 min), 8 °C/min 300 °C (3 min)	(1) EI 70 eV, FS 20–400 *m*/*z* (2) 40–500 *m*/*z*	Manual: NIST MS, Wiley libraries. Reference standards.	(**>30**) volatiles: alkanes, aldehydes, alkenes, alcohols, and aromatics (**50**) semi-volatiles: citrates, phthalates, adipates, phosphates, alkanes, aldehydes, and carboxylic acids	SML (R. 10/2011) TTC (Cramer) and ToxTree	[29]
Packaging for fruit purée and juice (PE and PA)	Migration: B, C, 40 °C, 10 days	UHPLC-QTOF-MS	Zorbax Eclipse Plus C8 (2.1 × 100 mm, 1.8 µm), 0.3 mL/min, 5 µL inj., 35 °C, H_2_O:MeOH (ammonium formate/formic), gradient, 20 min	ESI(+), DIA-MS^2^ (SWATH),	Automatic: SciexOS. Manual identification: ChemSpider, Metlin, Massbank, in silico fragmentation, and the scientific literature. Caprolactam reference standards.	(**26**) NIAS: caprolactam, bis(2-methoxyethyl) adipate, additives, and cyclic oligomers.	EU REACH-ECHA TTC (Cramer) and ToxTree	[30]
Pellets, containers, and films (PE)	Extraction: DCM, Soxhlet for 16 h, evaporation and reconstitution (ACN)	(1) Flow injection analysis (FIA)-MS. (2) FIA-HRMS (Q-Orbitrap)	FIA: 10 µL inj., flow 0.2 mL/min, elution solvent H_2_O:ACN 35:65 *v*/*v*, 0.1% formic acid (+) or 0.1% ammonia (-), 5 min	(1) ESI(+/−); FS 100–1200 *m*/*z*. (2) FS/MS^2^ 50–1500 *m*/*z*	Automatic: Matlab-Independent component analysis. Confirmation with Irganox 1010, Irganox 1076, Tinuvin 770, and Irgafos 168 standards.	(**8**) Irganox 1076, Irganox 1010, Irgafos 168, alkylbenzene sulfonates, phthalic anhydride, DBP, and DEHP.	-	[31]
Bottles, powders, and pellets (PET, rPET)	Total dissolution/precipitation: HFIP/MeOH	Direct sample introduction/GC × GC-HRMS (Q-TOF)	Column 1: DB-5MS, Column 2: DB-17MS (1 m × 0.25 mm × 0.25 µm), He 30 psi, 0.1 µL inj. 250 °C; 40 °C (5 min), 8 °C/min 300 °C (8 min)	EI 70 eV, FS 50–500 *m*/*z*	Manual: NIST MS library, and PubChem. Multivariate analysis: SIMCA, SPSS, and Matlab.	(**368**) 267 SVOCs, 41 (rPET), and 60 (vPET).	-	[32]
Kitchenwares (PA)	(1) Total dissolution/precipitation: HFIP/MeOH. (2) Migration: A, B, D2 (EtOH 95%), 70 °C, 2 h	UHPLC-QTOF-MS	BEH C18 (2.1 × 100 mm, 1.7 µm), 0.3 mL/min, 2 µL inj., 30 °C, H_2_O:MeOH (0.1% formic), gradient, 8 min	ESI(+/−); MS^E^, 50–1200 *m*/*z*	Automatic: UNIFI and a home-made database. Reference standards.	(**64**) phthalates, N-butyltri-n-hexyl citrate, antioxidants, slip agents, fatty acids TPP, PA, and PEG oligomers.	SML (R. 10/2011) TTC (Cramer) and ToxTree	[33]
Kitchenwares (melamine–formaldehyde resin)	Migration: D2 (EtOH 95%), 70 °C, 2 h	HPLC-QTOF-MS	Nova-Pak C18 (2 × 150 mm, 3 µm), 0.3 mL/min, 20 µL inj., 35 °C, H2O (0.1% formic):MeOH, gradient, 40 min	ESI(−), FS 100–600 *m*/*z*	Chemspider, PubChem, MassBank, and mzCloud. Melamine and formaldehyde standards.	Plasticizers, slip agents, surface-active agents, raw materials, processing aids, coating agents, and UV-protecting agents.	EDI and TDI (EFSA)	[34]
Films and rubbers (PP, PE, melamine resin, PC, PET, PS, PCT, ABS, TPU, PLA, acrylic resin, PBT, Fluororesin, PVC, PA, and PI)	Migration: n-heptane, D2 (EtOH 95%, isooctane), H_2_O, C, 4% acetic acid, 70 °C, 2 h	(1) UHPLC-QTOF-MS (2) GC-MS	1) Zorbax Eclipse Plus C18 (2.1 × 50 mm, 1.8 µm), 0.5 mL/min, 1 µL inj., 40 °C, H_2_O (0.1% formic): MeOH, gradient, 14 min 2) DB-5MS UI (30 m × 0.25 mm × 0.25 µm), 1 µm split 1:10, 280 °C, He (1 mL/min); 40 °C (3 min), 5 °C/min 300 °C (5 min)	(1) ESI(+/−); FS 100–1300 *m*/*z* (2) EI, 70 eV, FS 40–600 *m*/*z*	Automatic: MassHunter and the NIST MS library. Quantification with reference standards.	(1) (**69**) antioxidants, cyclic oligoesters, plasticizers, surfactants, emulsifiers, and flavor components. (2) (**22**) 1,3-ditert-butylbenzene, 2,4-di-tert-butylphenol, antioxidants phenylethyl alcohol, solvents, hydrocarbons, and styrene dimers.	Toxicity reference values (ECHA, NIH, and FDA) or TTC (Cramer)	[35]
Single-use and reusable containers (PP)	Migration: D2 (isooctane), 60 °C, 10 days	(1) GC-MS, (2) GC-TOF	1) DB-5MS UI (30 m × 0.25 mm × 0.25 µm), 1 µL SL 300 °C, He (2 mL/min); 40 °C (1 min), 25 °C/min 320 °C (10 min). 2) RTX-5MS (15 m × 0.25 mm × 0.25 µm), 1 µL split 1:50 250 °C, He (2 mL/min); 50 °C (1.5 min), 30 °C/min 320 °C (3 min)	EI, 70 eV, FS 50–700 *m*/*z*	Automatic: AMDIS and NIST MS. Confirmation with reference standards.	(**69**) 24 n-alkanes and 45 volatile and semi-volatile compounds.	-	[36]
Kitchenwares, disposable glasses, and reusable cups (SAN and ABS)	(1) Extraction: MeOH, ultrasounds, 50 °C, 30 min. (2) Migration: H_2_O, A, B, C, D1, 70/100 °C, 2 h	Multitechnique (HPLC-DAD/MS, HPLC-UV/Chemiluminescent nitrogen detector, HPLC-DAD/FLD, and GC-MS)	-	ESI(+), EI	Manual: The NIST MS library and the scientific literature. Confirmation with reference standards.	(**7**) 6 oligomers of styrene and acrylonitrile and primary aromatic amine.	TTC (Cramer) and ToxTree	[37]
Beverage can (Epoxy coating)	Extraction: ACN, 70 °C, 1 day	HPLC-MS/MS	Phenosphere ODS C18 (3.2 × 150 mm, 3 µm), 0.5 mL/min, 1 µL inj., 40 °C, H_2_O:ACN/MeOH, gradient, 28 min	APCI(+/−); FS 100–1000 *m*/*z*	Manual: A home-made database (BADGE derivatives and epoxy resin-related). Bisphenol-related standards.	(**10**) BADGE derivatives.	TTC (Cramer) and ToxTree	[38]
Pellets and bottles (PET and rPET)	Extraction: HS-SPME PDMS/DVB/Carbon WR,110 °C, 30 min; desorption: 250 °C, 2 min	HS-SPME-GC×GC-QTOF-MS	Column 1: HP-5MS (30 m × 0.25 mm × 0.25 µm). Column 2: DB-17MS (1 m × 0.25 mm × 0.25 µm), He 1.2 mL/min; 40 °C (5 min), 8 °C/min 260 °C (8 min)	EI 70 eV, FS 35–500 *m*/*z*	Automatic: MassHunter, the NIST MS library, RI, and ClassyFire	(**1247**) VOCs: hydrocarbons, benzenoids, organic oxygen compounds, lipids, and lipid-like compounds.	-	[39]
Coatings for metal cans (Epoxy coating)	(1) Extraction: ACN, room temp, 24 h (2) Migration: C, D1, 21 °C, 1 h	HPLC-TOF-MS	Atlantis dC18 (150 × 2.1 mm, 3 µm), 30 °C	ESI(+/−); FS 100–1100 *m*/*z*	Automatic: MassHunter, Mass Profiler Professional, and the V70 NIAS database (TMBPF-DGE oligomers). Semi-quantification with BADGE.	(**66**) 16 identified TMBPF-DGE oligomers and TMBPF-DGE + hydroquinone	Prioritization and hazard assessment using bioassays	[40]
Food metal cans (Epoxy coating)	Migration: A, 60 °C, 10 days	UHPLC-HRMS (Q-Orbitrap-LIT)	Hypersil Gold C18 (2.1 × 100 mm, 1.9 µm), 0.3 mL/min, 5 µL inj., H_2_O:MeOH (0.1% formic), gradient, 30 min	H-ESI(+), FS 100–900 *m*/*z* /MS^2^/MS^3^ LIT. AcquireX DeepScan mode	Automatic: Compound Discoverer, mzCloud, ChemSpider, and MassLists. Reference standards and semi-quantification (average response factor).	(**263**) 22 IAS/241 NIAS: polymer additives	SML (R. 10/2011) TTC (Cramer) and ToxTree	[41]
Film (rLDPE)	Extraction: ACE, 40 °C, 1 h	GC-HRMS (Q-Orbitrap)	TG-5MS (30 m × 0.25 mm, 0.25 µm), 1 µL SL 280 °C, He (1.2 mL/min); 40 °C (5 min), 5 °C/min 315 °C (10 min)	EI 70 eV, FS 40–500 *m*/*z*	Automatic: Compound Discoverer, the NIST MS library, RI, and GC Orbitrap databases. Reference standards and semi-quantification (average response factor).	(**83**) 12 IAS/71 NIAS: additives, metabolites, industrial compounds, and PE oligomers	SML (R. 10/2011) TTC (Cramer) and ToxTree	[42]
Baby bottles (PP, Tritan, and silicone)	Migration: D1, 70 °C, 2 h	UHPLC-QTOF-MS	UPLC BEH C18 (2.1 × 100 mm, 1.7 µm), 0.3 mL/min, 10 µL inj., 40 °C, H_2_O:MeOH (0.1% formic), gradient, 10 min	ESI(+/−); 50–1000 *m*/*z*, MS^E^	Automatic: MassLynx. Manual: ChemSpider, SciFinder, and MassFragment. Reference standards and external calibration.	(**27**) 2.2′-(tridecylimino)bis-ethanol and derivatives, clarifying agents, glycerol derivatives, erucamide, and N-acetylvaline	SML (R. 10/2011) TTC (Cramer) and ToxTree	[43]
Metallic FCMs (PES-PUR coatings)	Extraction: ACN, 40 °C, 24 h, evaporation and reconstitution	UHPLC-HRMS (Q-Orbitrap)	Hypersil Gold C18 (2.1 × 100 mm, 1.9 µm), 0.4 mL/min, 10 µL inj., 40 °C, H_2_O:ACN (10 mM ammonium acetate), gradient, 20 min	H-ESI(+,−); FS 155–1200 *m*/*z*, targeted-MS^2^ (22 precursors)	Automatic: the R environment and a home-made database of predicted oligomers.	(**58**) 28 predicted oligomers and 26 unpredicted monomers and oligomers	SML (R. 10/2011)	[44]
Metallic FCMs (PES-PUR coatings)	Extraction: ACN, 40 °C, 24 h, evaporation and reconstitution	Multitechnique (GC-EI-MS, GC-Q-Orbitrap, GC-APCI-TOF-HRMS, and GC × GC-EI-TOF-MS)	-	-	Manual: The NIST MS library.	isophorone diisocyanate, 4,4-diphenylmethane diisocyanate, and cyclic oligoesters	-	[45]
Pellets (Bioplastics)	Extraction: n-Hexane:ACE (1:1), room temp, overnight, evaporation and reconstitution	UHPLC-HRMS (Q-Orbitrap)	Acquity BEH C8 (2.1 × 100 mm, 1.7 µm), 0.4 mL/min, 5 µL inj., 40 °C, H_2_O (formic/formate):MeOH, gradient, 33 min	H-ESI(+); FS 80–1200 *m*/*z*, DDA-MS^2^	Manual: The Xcalibur Qual Browser. Automatic: Progenesis QI and MN using Python and Cytoscape.	(**96**) PLA, PBAT, and PBS oligomers	-	[46]
Powder, pellets, and retail samples (PLA and starch biopolymers)	(1) Extraction: MeOH, 40 °C, 1 h, evaporation. (2) Migration: A, B, D2 (EtOH 95%), 70 °C, 6 h	(1) GC-EI-MS; (2) APGC-QTOF-MS	HP-5MS (30 m × 0.25 mm, 0.25 µm), 1 µL SL 250 °C, He 1 mL/min; 50 °C (5 min), 10 °C/min 300 °C (5 min)	(1) EI 70 eV, FS 50–450 *m*/*z*; (2) APCI(+), 50–650 *m*/*z*, MS^E^	Automatic: MSD ChemStation, the NIST MS library, and UNIFI. Reference standards and external calibration.	(**21**) 14 identified antioxidants, lubricants, fatty acids, alcohols, slip agents, plasticizers, glucitol, and mono-2-ethyloxoexyl adipate	TTC (Cramer)	[47]
Pristine, contaminated, washed, and/or recycled pellets (rPP)	Extraction: HS-SPME (DVB/CAR/PDMS), 120 °C, 30 min	HS-SPME-GC-Olfactometry (O)-MS	HP-5MS (30 m × 0.25 mm, 0.25 µm), SPME desorption 250 °C, 2 min; He 1 mL/min; 40 °C (5 min), 10 °C/min 300 °C (2 min). Olfactometry with 3 trained panelist	EI, FS 45–350 *m*/*z*	Manual: The NIST MS Library, the scientific literature, and the FlavorDB, Pherobase, Flavornet databases.	(**45**) IAS: toluene, benzophenone, tetracosane; NIAS: glycerin, alkanes, and alkenes	-	[48]
Pellets and films (LDPE)	-	Raman spectroscopy; Micro-Raman imaging; ICP-MS (Ca and Ti)	-	-	Identification and quantification using of Ca and Ti by ICP-MS.	CaCO_3_, CaSO_4_, PS, and TiO_2_	TTC (Cramer)	[49]
Multilayer films (LDPE and rLDPE)	(1) Extraction: Soxhlet, CHCl_3_, 60 °C, 5 h. (2) Migration: D2 (EtOH 95%), 20–60 °C, 10 days	HPLC-DAD; GC-MS	(1) HPLC: ZORBAX Eclipse XDB-C18, 10 µL, 1 mL/min. (2) GC: ZB-5MSPlus (Phenomenex, Torrance, CA, USA) (30 m × 0.25 mm × 0.25 mm), split (2:1 ratio), 60 °C to 300 °C at 10 °C/min	-	Confirmation and quantification using commercial standards.	Antioxidants, 2,4-di-tert-butylphenol (arvin 4), arvin 8, DEHP, DEHTP, and oligomers	SML (R. 10/2011)	[50]
Recyclates (rHDPE, rLDPE, rPE, rPET, rPP, and rPS)	Extraction: DCM, 40 °C, 3 days (polyolefins, PET); ACE, 60 °C, 3 days (PS)	Multitechnique (GC-FID; GC-MS; HS-GC-MS; HPLC-MS)	-	EI, FS 40–800 *m*/*z*; ESI, FS 500–1200 *m*/*z*	Manual: The NIST MS Library and retention time comparison with internal standards. Quantification of Irgafos 168, oxidized Irgafos 168, Irganox 245, Irganox 1010, Irganox 1076, Irganox 1330, and limonene	(**205**) 175 tentatively identified and 30 unknowns: alkanes, plasticizers, thermal stabilizers, flame retardants, antioxidants, and light and heat stabilizers.	TTC (Cramer) and (Non)-Genotoxic Carcinogenicity Alert	[51]
Microwave trays and bags and oven bags	Migration: Microwave, D2 (95% EtOH), 102 °C, 5 min; Oven, D2 (95% EtOH), 60 °C, 6 h	(1) GC-HRMS (Q-Orbitrap) (2) UHPLC-HRMS (Q-Orbitrap)	(1) GC: TG-5SilMS (30 m, 0.25 mm, 0.25 µm), 1 µL SL, He 1 mL/min, 50 °C (2 min), 20 °C/min 150 °C, 6 °C/min 320 °C (10 min); (2) LC: C18 (100 × 1 mm × 1.7 µm), inj. 2 µL, 45 °C, 60 µL/min, H_2_O:ACN gradient 35 min	(1) GC: EI 70 eV, FS 50–750 *m*/*z* (2) LC: H-ESI(+/−); FS/ddMS^2^ 150–800 *m*/*z*	Automatic: Compound Discoverer, the NIST MS Library, GC-Orbitrap libraries; mzCloud, the Extractables and Leachables HRAM database, and ChemSpider.	(**74**) 65 IAS and 9 NIAS	-	[52]
Containers (expanded PS and rEPS)	(1) Extraction: HS-SPME, 30–50 μm DVB/CAR/PDMS, 100 °C (qual)/85 °C (quant), 15 min; desorption: 250 °C, 2 min. (2) Migration: A, B, 60 °C, 10 days	HS-SPME-GC-MS; SPME-GC-MS	HP-5MS (30 m × 0.25 mm, 0.25 µm), SL 250 °C, He 1 mL/min; 50 °C (5 min), 10 °C/min 300 °C (5 min)	EI, FS 45–400 *m*/*z*	Statistical analysis: SIMCA. Validation with reference standards of ethylbenzene, o-xylene, styrene, and 1,4-diphenylbutane.	(**99**) hydrocarbons, aldehydes, ketones, alcohols, esters, benzene derivatives, aromatics, styrene dimmers, and additives	SML (R. 10/2011) TTC (Cramer) and ToxTree	[53]
Sheets (PP)	Extraction: DCM, ultrasounds, 1 h, evaporation	(1) EI-GC-MS (2) APCI-QTOF-MS	(1) GC-MS: HP-5MS (30 m × 0.25 mm, 0.25 µm), 2 µL SL 250 °C, He 2.4 mL/min; 50 °C (3 min), 10 °C/min 300 °C (12 min) (2) APCI-QTOF-MS: SPB 5 (30 m × 0.25 mm, 0.25 µm), 2 µL SL 250 °C, He 3.5 mL/min; 50 °C (3 min), 10 °C/min 300 °C (12 min)	(1) GC-MS: EI, FS 40–700 *m*/*z* (2) QTOF-MS: 40–700 *m*/*z*, MS^E^	Manual: Masslynx and AMDIS from NIST. Confirmation with analytical standards.	(**27**) antioxidants, lubricants, catalysts, and transformation products	-	[54]
Can coatings (PES-phenolic resole-based resins)	Extraction: ACN, 40 °C, 24 h	(1) GC-MS (2) GC-HRMS (Q-Orbitrap)	(1) GC-MS: HP-5MS (30 m × 0.25 mm, 0.25 µm), 1 µL SL 280 °C; He 1 mL/min; 80 °C (2 min), 10 °C/min 270 °C (20 min), 5 °C/min 320 °C (15 min) (2) GC-HRMS: TG-5SilMS (30 m × 0.25 mm, 0.25 µm), 1 µL SL 280 °C; He 1 mL/min; 80 °C (4 min), 10 °C/min 320 °C (15 min)	(1) GC-MS: EI 70 eV, FS 50–750 *m*/*z* (2) GC-HRMS: EI 10 and 70 eV, FS 50–750 *m*/*z*	Manual: NIST MS and Wiley libraries and a home-made oligomers database. Semi-quantification of phenol-based molecules and PES oligomers. Diethyl terephthalate as the internal standard.	Cyclic PES oligomers and aldehydes	-	[55]
Reusable bottles (Unknown, PP, and Tritan)	Migration: D1, 40 °C, 10 days	HPLC-QTOF-MS	Poroshell 120 Phenyl Hexyl (2.7 μm × 3.0 mm×100 mm) with Poroshell 120 EC-C18 (2.7 μm × 3.0 mm×10 mm) guard column; 10 µL inj., 20 °C, 0.2 mL/min; H_2_O:MeOH (0.1% formic); gradient, 20 min	All ions mode (MS^2^), 50–1700 *m*/*z*	Automatic: MassHunter Profiling and Agilent Extractables and Leachables LC/QTOF databases. Analytical standards.	Monomethyl terephthalate	-	[56]
Reusable bottles (PE and biodegradable PE)	Migration: drinking water stored for 24 h and SPE	UHPLC-QTOF-MS	Acquity BEH C18 (2.1 × 100 mm, 1.7 µm), 0.3 mL/min, 2 µL inj., 40 °C, H_2_O:ACN (0.1% formic), gradient, 24 min	ESI(+), MS^E^ and MS^2^ 50–1200 *m*/*z*	Automatic: UNIFI with Norman lists “database of chemicals associated with plastic (CPPdb)” and “plastic additives by ECHA, PubChem, MassBank”.	(**>3500**) oligomers, aromatic amines, plasticizers, antioxidants, and photoinitiators	TTC (Cramer) and ToxTree	[57]
Reusable bottles (Silicone, HDPE, LDPE, PP, PS, PET, PETG, and PCTG)	Migration: tap water at room temp., 48 h, evaporation and reconstitution (MeOH)	UHPLC-QTOF-MS	BEH C18 (2.1 × 100 mm, 1.7 µm), 0.3 mL/min, 1 µL inj., H_2_O:ACN (0.1% formic), gradient, 16 min	DIA-MS^E^, ESI(+/−), 50–1000 *m*/*z*	Manual: MSDial. Quantitative non-target analysis (qNTA) employing QSPR. Analytical standards of six model additives.	Phthalates, color agents, sorbitol-based nuclear clarifying agents, BADGE derivates, intermediates, plasticizer, and oligomers	TTC (Cramer)	[58]
Pellets, bottles (PET and rPET)	(1) Total dissolution/precipitation: HFIP, 40 °C, 24 h; MeOH, 4 °C, 1 h (2) Extraction: DCM, ultrasounds, 1 h, evaporation and reconstitution (MeOH) (3) Migration: A, B, D2 (95% EtOH), 60 °C, 10 days	UHPLC-QTOF-MS	BEH C18 (2.1 × 100 mm, 1.7 µm), 0.3 mL/min, 10 µL inj., 35 °C, H_2_O:MeOH (0.1% formic), gradient, 8 min	ESI(+/−), MS^E^	Automatic: MassLynx, MassFragment. Quantification with the oligomer AA-DEG-IPA-DEG.	PET cyclic and linear oligomers	-	[59]
Pellets and films (PLA and PES biopolymers)	(1) Total dissolution/precipitation: DCM, ultrasounds, 1 h; EtOH, 4 °C, 1 h. (2) Migration: A, B, D2 (95% EtOH), 60 °C, 10 days	UHPLC-QTOF-MS, UHPLC-IMS-QTOF-MS	BEH C18 (2.1 × 100 mm, 1.7 µm), 0.3 mL/min, 10 µL inj., 40 °C, H_2_O:MeOH (0.1% formic), gradient, 12 min	ESI(+/−), MS^E^	Automatic: MassFragment and ChemDraw Ultra. Quantification with the oligomer AA-DEG-IPA-DEG.	(**39**) PLA cyclic and linear oligomers	-	[60]
Pellets and films (PLA and PES biopolymers)	(1) Total dissolution/precipitation: DCM, ultrasounds, 1 h; EtOH, 4 °C, 1 h. (2) Migration: A, B, D2 (95% EtOH), 60 °C, 10 days	(1) GC-MS (2) APGC-QTOF-MS (3) GC-O-MS	(1,3) GC-(O)-MS: HP-5MS (30 m × 0.25 mm × 0.25 μm), HS-SPME, He 1 mL/min, 40 °C (5 min), 10 °C/min 300 °C (2) APGC-QTOF-MS: HP-5MS (30 m × 0.25 mm × 0.25 μm), 1 µL SL 250 °C, He 1.2 mL/min, 60 °C (5 min), 10 °C/min 300 °C (5 min)	GC-MS: EI-MS; APGC-QTOF-MS: API(+), MS^E^, 50–550 *m*/*z*	Automatic: MassLynx. Quantification using a cyclic ester oligomer, octanal, 1-octen-3-one, (E)-2-nonenal, sotolon, citronellal, dodecanal, and nonanal as standards.	(**15**) lactide, cyclopentanone, cyclic dimer, adipic acid, butanediol, palmitic acid, oleamide, glycerol 1-palmitate, glycerol 1-stearate, and erucamide	-	[61]
Bottles and pellets (rPET)	(1) Extraction: DCM, 40 °C, 3 days. (2) Migration: H_2_O, 40 °C, 10 days	HS-GC-MS, GC-MS, GC-FID	DB1 MS (30 m × 0.25 mm × 0.25 μm), various temperature programs and conditions	EI, FS 40–800 *m*/*z*	Manual: The NIST MS library and AKTS SML for migration modeling. Quantification with analytical standards.	Acetaldehyde, ethylene glycol, 2-methyl-1,3-dioxolane, limonene, acetone, butanone, furan, benzene, styrene, and oligomers	SML (R. 10/2011) TTC (Cramer)	[62]
Films (PP)	Migration: A, B, D2 (95% EtOH), E, 60 °C, 10 days	UHPLC-QTOF-MS	BEH C18 (2.1 × 100 mm, 1.7 µm), 0.3 mL/min, 5 µL inj., 35 °C, H_2_O:MeOH (0.1% formic), gradient, 15 min	ESI(+/−), MS^E^	Automatic: MassLynx, CromaLynx, and MassFragment. Quantification with analytical standards.	(**76**) Irganox 1076 and Irganox 1010 degradation products or impurities	SML (R. 10/2011) TTC (Cramer)	[63]
Films (HDPE and LDPE)	Migration: A, B, D1, D2 (95% EtOH), E, 60 °C, 10 days	UHPLC-IMS-QTOF-MS	BEH C18 (2.1 × 100 mm, 1.7 µm), 0.3 mL/min, 5 µL inj., 35 °C, H_2_O:MeOH (0.1% formic), gradient, 13 min	ESI(+/−), high definition MS^E^	Automatic: UNIFI and ChemSpider. Quantification with analytical standards.	(**35**) Irganox 1010 and Irganox 1076 degradation, breakdown, impurity, or reaction products	SML (R. 10/2011) TTC (Cramer)	[64]
Bags and tetrabrik (PP and PE)	Migration: E, 60 °C, 10 days	GC-O-MS	BP-20 (30 m × 0.25 mm × 0.25 μm), HS-SPME, He 1 mL/min, 40 °C (5 min), 10 °C/min 220 °C (10 min)	FS/SIM, 50–450 *m*/*z*	Manual: NIST and Wiley MS libraries. Quantification and confirmation with analytical standards.	(**46**) acetic, propanoic, butyric acid, octanal, nonanal, decanal, trimethylbenzenes, and terpenes	SML (R. 10/2011) TTC (Cramer)	[65]
Corks (EVA)	Migration: B, C, 60 °C, 10 days	(1) GC-MS (2) UHPLC-IMS-QTOF-MS	(1) GC-MS: HP-5 MS (30 m × 0.25 mm × 0.25 μm), SPME, He 1 mL/min, 50 °C (5 min), 10 °C/min 300 °C (5 min). (2) UHPLC-IMS-QTOF-MS: BEH C18 (2.1 × 100 mm, 1.7 µm), 0.3 mL/min, 5 µL inj., 40 °C, H_2_O:MeOH (0.1% formic), gradient, 13 min	(1) GC-MS: FS, 50–450 *m*/*z*; (2) UHPLC-IMS-QTOF-MS: 50–1000 *m*/*z*, high definition MS^E^	Automatic: UNIFI, Chemspider, NIST, and Wiley MS libraries. Quantification and confirmation with analytical standards.	(**50**) antioxidants, lubricants, cyclic oligomers, and breakdown and oxidation products	SML (R. 10/2011) TTC (Cramer)	[66]
Post-consumer film (rLDPE)	Migration: A, 40 °C, 10 days	UHPLC-HRMS (Q-Orbitrap-LIT)	Hypersil Gold C18 (2.1 × 100 mm, 1.9 µm), 0.3 mL/min, 5 µL inj., 40 °C, H_2_O:MeOH, gradient, 30 min	H-ESI(+/−), FS 100–900 *m*/*z*, MS^2^/MS^3^	AcquireX DeepScan and Interative Processing Exclusion. Automatic: Compound Discoverer, mzCloud, and ChemSpider.	(**28**) additives and plasticizers	SML (R. 10/2011) TTC (Cramer)	[67]
Bowls (PC)	Migration: C, 100 °C, 2 h	UHPLC-HRMS (Q-Orbitrap-LIT)	Hypersil Gold C18 (2.1 × 100 mm, 1.9 µm), 0.3 mL/min, 5 µL inj., 40 °C, H_2_O:MeOH, gradient, 30 min	H-ESI(+/−), FS 100–900 *m*/*z*, MS^2^/MS^3^	AcquireX DeepScan and Interative Processing Exclusion. Automatic: Compound Discoverer, mzCloud, and ChemSpider. Analytical standards.	(**24**) plasticizers, slip agents, antioxidants, UV stabilizers, and fragrances	SML (R. 10/2011) TTC (Cramer)	[68]
Bowls and cups (PPCs)	Extraction: EtOH, isooctane, 4% acetic acid, 40–60 °C, 1–2 h	UHPLC-QTOF-MS	Zorbax SB C18 (2.1 × 100 mm, 1.8 µm), 0.3 mL/min, 3 µL inj., 40 °C, H_2_O (0.1% formic):MeOH, gradient, 35 min	ESI(+/−), DDA-MS^2^, 50–1000 *m*/*z*	Automatic: MS-DIAL, MS-FINDER, NIST, MoNA, and GNPS libraries.	(**115**) plasticizers, pesticides, bisphenols, oligomers, and melamine derivatives	SML (R. 10/2011) TTC (Cramer)	[69]

## Data Availability

No new data were created or analyzed in this study. Data sharing is not applicable to this article.

## Abbreviations

The following abbreviations are used in this manuscript:

AA	Adipic acid
ABS	Acrylonitrile–butadiene–styrene
ACE	Acetone
ACN	Acetonitrile
AMDIS	Automated Mass spectral Deconvolution and Identification System
APCI	Atmospheric pressure chemical ionization
AZA	Azelaic acid
BADGE	Bisphenol A diglycidyl ether
BHET	Bis(2-hydroxyethyl) terephthalate
BHT	Butylated hydroxytoluene
bw	Body weight
CAR	Carboxen
CCS	Collision cross-section
CE	Collision energy
CF	Consumption factor
DAD	Diode array detector
DBP	Dibutyl phthalate
DCM	Dichloromethane
DDA	Data-dependent acquisition
ddMS^2^	Data-dependent tandem mass spectrometry
DEG	Diethylene glycol
DIA	Data-independent acquisition
DMSO	Dimethyl sulfoxide
DVB	Divinylbenzene
EDI	Estimated daily intake
EFSA	European Food Safety Authority
EI	Electron ionization
EPA	U.S. Environmental Protection Agency
EPS	Expanded polystyrene
ESI	Electrospray ionization
EtOH	Ethanol
EVA	Ethylene–vinyl acetate
FCMs	Food contact materials
FDA	U.S. Food and Drug Administration
FIA	Flow injection analysis
FID	Flame ionization detector
FLD	Fluorescence detector
FS	Full scan
FWHM	Full width at half maximum
GC	Gas chromatography
GC×GC	Two-dimensional gas chromatography
HDPE	High-density polyethylene
HFIP	Hexafluoroisopropanol
HPLC	High-performance liquid chromatography
HQ	Hazard quotient
HRMS	High-resolution mass spectrometry
HS	Headspace
IAS	Intentionally added substances
ICP-MS	Inductively coupled plasma mass spectrometry
IMS	Ion mobility spectrometry
IPA	Isophtalic acid
LC	Liquid chromatography
LDPE	Low-density polyethylene
LIT	Linear ion trap
MeOH	Methanol
MN	Molecular networking
MS	Mass spectrometry
MS/MS	Tandem mass spectrometry
NIAS	Non-intentionally added substances
NIST	National Institute of Standards and Technology
NMR	Nuclear magnetic resonance
NTA	Non-targeted analysis
PA	Polyamide
PBAT	Polybutylene adipate terephthalate
PBS	Polybutylene succinate
PBT	Polybutylene terephthalate
PC	Polycarbonate
PCT	Polycyclohexane-1,4-dimethylene terephthalate
PCTG	Polycyclohexylenedimethylene terephthalate glycol-modified
PDMS	Polydimethylsiloxane
PE	Polyethylene
PES	Polyester
PET	Polyethylene terephthalate
PETG	Polyethylene terephthalate glycol-modified
PI	Polyimide
PLA	Polylactic acid
PP	Polypropylene
PPCs	Plant fiber/plastic composites
PPSU	Polyphenylsulfone
PS	Polystyrene
PUR	Polyurethane
PVC	Polyvinyl chloride
P&T	Purge and trap
qNTA	Quantitative non-target analysis
QSPR	Quantitative structure-property relationship
QTOF	Quadrupole time-of-flight
RI	Retention index
rEPS	Recycled expanded polystyrene
rHDPE	Recycled high-density polyethylene
rLDPE	Recycled low-density polyethylene
rPE	Recycled polyethylene
rPET	Recycled polyethylene terephthalate
rPP	Recycled polypropylene
rPS	Recycled polystyrene
SAN	Styrene-acrylonitrile copolymer
SML	Specific migration limit
SPME	Solid-phase microextraction
SVOCs	Semi-volatile organic compounds
SWATH	Sequential windowed acquisition of all theoretical MS
TDI	Tolerable daily intake
TMBPF-DGE	Tetramethyl bisphenol F-based diglycidyl ether
TPU	Thermoplastic polyurethane
TTC	Threshold of toxicological concern
UHPLC	Ultra-high-performance liquid chromatography
UV	Ultraviolet radiation
VOCs	Volatile organic compounds
WHO	World Health Organization
